# Loss of function of myosin chaperones triggers Hsf1-mediated transcriptional response in skeletal muscle cells

**DOI:** 10.1186/s13059-015-0825-8

**Published:** 2015-12-03

**Authors:** Christelle Etard, Olivier Armant, Urmas Roostalu, Victor Gourain, Marco Ferg, Uwe Strähle

**Affiliations:** Institute of Toxicology and Genetics, Karlsruhe Institute of Technology, Campus Nord, PO box, Karlsruhe, Germany; Present address: Institute of Inflammation and Repair, Michael Smith Bldg, University of Manchester, Oxford Road, Manchester, M13 9PL UK

**Keywords:** unc45b, Myosin chaperones, hsf1

## Abstract

**Background:**

Mutations in myosin chaperones Unc45b and Hsp90aa1.1 as well as in the Unc45b-binding protein Smyd1b impair formation of myofibrils in skeletal muscle and lead to the accumulation of misfolded myosin. The concomitant transcriptional response involves up-regulation of the three genes encoding these proteins, as well as genes involved in muscle development. The transcriptional up-regulation of *unc45b*, *hsp90aa1.1* and *smyd1b* is specific to zebrafish mutants with myosin folding defects, and is not triggered in other zebrafish myopathy models.

**Results:**

By dissecting the promoter of *unc45b*, we identify a Heat shock factor 1 (Hsf1) binding element as a mediator of *unc45b* up-regulation in myofibers lacking myosin folding proteins. Loss-of-function of Hsf1 abolishes *unc45b* up-regulation in mutants with defects in myosin folding.

**Conclusions:**

Taken together, our data show that skeletal muscle cells respond to defective myosin chaperones with a complex gene program and suggest that this response is mediated by Hsf1 activation.

**Electronic supplementary material:**

The online version of this article (doi:10.1186/s13059-015-0825-8) contains supplementary material, which is available to authorized users.

## Background

Formation of the contractile myofibril of the skeletal muscle is a complex process and involves the correct synthesis, folding and assembly of a huge number of proteins. Auxiliary proteins such as Unc45b or Hsp90aa1.1 (referred to as Hsp90a) contribute to this process by folding the myosin motor domain and organizing the filament structure [1–4]. Animals homozygous for loss-of-function mutations in these myosin folding genes fail to assemble myofibrils and are totally paralyzed [1, 5–12]. A similar phenotype was recently observed in zebrafish carrying mutations in the methyl transferase *smyd1b* [13–15]. In zebrafish, *unc45b*, *hsp90a* and *smyd1b* are specifically expressed in cardiac and skeletal muscle [1, 9, 13]. Pull-down experiments suggest that Hsp90a and Unc45b form a complex with nascent myosin [10, 11]. Although it has not been shown to be directly a chaperone, Smyd1b is pivotal for proper thick filament assembly and interacts with both Unc45b and myosin [13–15].

Unc45b is composed of an N-terminal tetratricopeptide repeat (TPR) domain implicated in binding the Hsp90a partner [11], a central armadillo repeat (ARM) domain with presumptive dimerization function and a C-terminal UCS domain required to interact with the motor domain of myosin [16, 17]. UCS domain-containing genes were found in organisms as diverse as yeast and human. Vertebrates have two Unc45 paralogs. Unc45a has been shown to cooperate with Hsp90 in chaperoning mammalian progesterone receptor [18] and plays a role in pharyngeal and aortic development in zebrafish [19]. Unc45b was proposed to be required for the folding of myosins in general, including those myosins that are not part of the myofibril [20, 21]. Missense mutations in *unc45b* have been associated with juvenile cataract in humans, a phenotype that is also evident in the *unc45b* zebrafish mutant [22]. Indeed, in addition to the strong expression in the musculature noticed previously [1], low level expression of *unc45b* was also detected in the lens and the retina [22]. Unc45b may also have roles other than myofibrilogenesis: Unc45b was shown to interact with the C to U deaminases Apobec2a/b in zebrafish. Knockdown of Unc45b and Apobec2 proteins present a muscular dystrophy-like phenotype in the zebrafish embryo [23].

In zebrafish, both Unc45b and Hsp90a are transiently enriched at the nascent A-band, but are kept at the Z-line in the mature fiber [12]. Sarcolemmal lesions in mature fibers trigger prompt relocalization of the chaperones to the A-band [12]. This suggests that the Z-line serves as a reservoir of chaperones for rapid recruitment to sites of myosin assembly. Myosin folding and thick filament assembly play an important role throughout the life of a vertebrate. The contractile apparatus is subject to rapid turnover depending on nutrition, exercise and health status of the animal [24]. Thus, the auxiliary chaperones involved in myosin folding need to be present at sufficient levels to achieve efficient muscle remodeling. However, too much Unc45b appears to be detrimental to the cell [25]. Transgenic worms overexpressing UNC-45 display defects in myosin assembly and a mild paralysis phenotype [6]. Aberrant stabilization of Unc45b protein by mutations in the ubiquitin ligase Chip causes muscle defects in worms [26] and mutations in the human homolog of Chip were identified as causes of late onset inclusion body myopathy [27]. Loss-of-function mutations in any one of the known myosin folding genes — *unc45b*, *hsp90a* or the myosin chaperone complex partner *smyd1b* — cause an increase in their own expression [1, 9, 13]. This suggests that muscle cells regulate Unc45b at multiple levels, including subcellular localization, protein stability and mRNA expression.

We report here the investigation of the mechanisms underlying the up-regulation of the mRNA of *unc45b*. Our results suggest that the increase in its expression is linked to the failure to fold myosin and is not a general response to paralysis or defective myofibrils. We analyzed the changes of the transcriptome in *unc45b* and *hsp90a* mutants. Defective myosin folding leads to a complex transcriptional response, including both chaperones as well as proteins involved in muscle and cardiac development. To elucidate the mechanism, we established an *unc45b* promoter-based transgene model and mapped the response to a heat shock element in the 5’ region of the *unc45b* gene. Knock-down of Heat shock factor 1 (Hsf1) abolished the upregulation of *unc45b* mRNA. Taken together, our work reveals a complex transcriptional response to impaired myosin folding that involves Hsf1 as a mediator and presumably also as a sensor of the accumulation of misfolded myosin.

## Results

### Up-regulation of myosin chaperones is specific to mutants with myosin folding defects

**Fig. 1:**
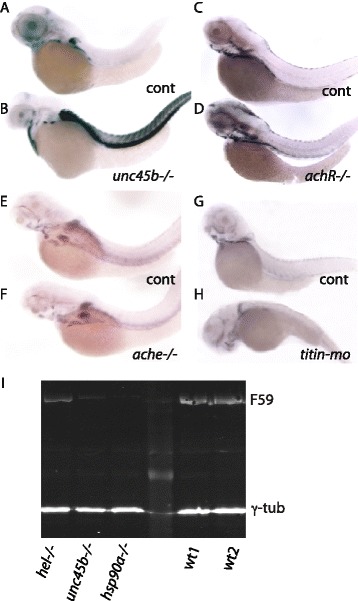
Expression of *unc45b* mRNA in muscle mutants. *unc45b* mRNA expression in wild-type sibling (**a**) and *unc45b* mutant (**b**) embryos. *unc45b* mRNA expression in wild-type sibling (**c**) and *sop* mutant (**d**) embryos with a defective delta subunit of acetylcholine receptor. *unc45b* mRNA expression in wild-type sibling (**e**) and *ache* mutant (**f**) embryos encoding a defective acetylcholine esterase. *unc45b* mRNA expression in control (**g**) and *titin* morphant (**h**) embryos. With the exception of *unc45b* mutants (**b**) with a myosin folding defect, none of the other mutants with impaired muscle function (**d**, **f**, **h**) showed up-regulation of *unc45b* mRNA. Embryos were hybridized to *unc45b* antisense RNA. All embryos are 72 h old; anterior left, dorsal up. **i** Myosin content in different mutants compared with wild type. Western blot done with protein extracts from embryos 72 hours post-fertilization: titin mutant (*hel−/−*), *unc45b* mutant (*unc45b−/−*), *hsp90a* mutant (*hsp90a−/−*) and wild type (*WT*). Antibodies: F59 recognizing slow myosin, and γ-tubulin as a loading control

Impaired formation of myofibrils in zebrafish with mutations in the *unc45b*, *hsp90a* and *smyd1b* genes is associated with increased abundance of the transcripts of the three genes in the muscle [1, 9, 13]. In addition to the lack of striated myofibrils, the three mutants are characterized by paralysis and in the chaperone mutants also by the presence of aggregates of misfolded myosin in the cytoplasm [1, 9, 13]. To test whether paralysis is the cause of these transcriptional responses, we analyzed several other mutants and morphants with impaired function or formation of the muscle by in situ hybridization against the target genes. *sop*^*fixe*^ mutants carry a mutation in the delta subunit of the acetylcholine receptor, are completely paralyzed and form slightly thinner but normally striated muscle fibers [28]. In comparison with *unc45b* mutants (Fig 1a, b), the myofibers of *sop*^*fixe*^ mutants (*achR−/−*) do not show elevated levels of *unc45b* mRNA (Fig. 1c, d). *ache*^*sb55*^ mutants lack functional acetylcholine esterase and develop progressive muscle degeneration [29]. *Ache* mutants did not elicit an up-regulation of *unc45b* mRNA expression (Fig. 1e, f). Similarly, in *titin* morphants in which sarcomers are disorganized, *unc45b* expression remained at the same level as in uninjected control larvae (Fig. 1g, h). This result was confirmed by analysis of the *titin* mutant *herzschlag* (*hel*), exhibiting no up-regulation of *unc45b* and *hsp90a* transcripts (data not shown) at 24 or 36 hours post-fertilization (hpf) in contrast to *unc45b* mutants [30]. Comparable results were obtained when these mutants and morphants were hybridized with antisense RNA directed against *smyd1b* or *hsp90a* mRNA (Fig. 1g, h and data not shown). Thus, neither paralysis nor disorganized myofibrils per se appear to trigger the elevated expression of myosin folding genes in *unc45b* mutants.

*unc45b−/−*, *hsp90a−/−* and *smyd1b−/−* mutants express lower levels of myosin [9, 13, 25] (Fig. 1i). We performed western blot analysis with protein extracts from 72-hpf mutant embryos. Like *unc45b−/−* and *hsp90a−/−* mutants, *hel−/−* mutants show approximately 50 % lower levels of slow muscle myosin expression than wild-type embryos (Fig. 1i), but do not show up-regulation of *hsp90a* or *unc45b* mRNA expression (Fig. 1g, h and data not shown). Reduction of myosin levels by knock-down of the major muscle myosin Myhc4 using a crispr/cas9 approach also did not elicit up-regulation of *unc45b* mRNA, although it strongly reduced the birefringence of the somites (data not shown). We thus conclude that the reduced expression of skeletal muscle myosins in the mutants is unlikely a cause of the up-regulation of the proteins involved in myosin folding. Taken together, these data rather suggest that the unfolded myosins may be the trigger for the up-regulation of the myosin chaperones.

### *unc45b* mutants activate complex gene expression programs

To address whether the increased expression is restricted to the genes encoding proteins responsible for myosin folding (*unc45b*, *hsp90a*), we analyzed the changes in the transcriptome of *unc45b* mutant and wild-type 72-hpf embryos. We chose 72 hpf because this is the time point showing the biggest difference in gene expression between wild type and *unc45b* mutants. RNA-Seq libraries were prepared from two biological replicates from total RNA and sequenced at a depth of at least 60 million 50-bp long paired-end reads per sample (Additional file 1). Data were subjected to normalization and quality control (Figure. S1a in Additional file 2).

In total, 1411 genes were differentially expressed with a fold change of at least 1.5-fold (false discovery rate (FDR) < 0.05), including both up- and down-regulated genes in the mutant (Additional file 3). Hence, the response to defective *unc45b* appears to entail robust and comprehensive changes in the transcriptional activity of the muscle cell.

**Fig. 2:**
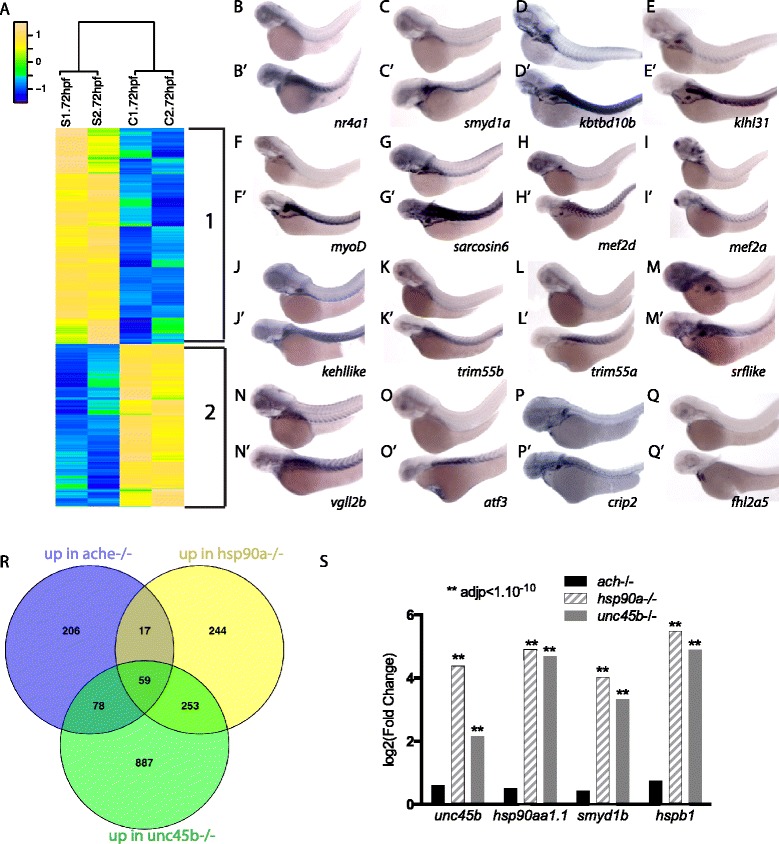
Transcriptome analysis of *unc45b* mutants. **a** Hierarchical clustering of genes significantly up-regulated (*yellow*) or down-regulated (*blue*) (fold change of at least 1.5-fold, p_adj_ < 0.05) in two independent mRNA samples from *unc45b* ^*−/−*^ embryos (*S1* and *S2*) relative to two mRNA preparations from wild-type siblings (*C1* and *C2*) at 72 hpf. The relative expression scale is indicated as normalized expression. Both up-regulated genes (851 genes, cluster 1) and down-regulated genes (560 genes, cluster 2) are shown. The relative expression scale is indicated as normalized expression (*blue* indicates low expression, *green* moderate expression, and *yellow* high expression). **b**–**q’** RNA in situ hybridization to verify RNAseq data. Genes targeted by the antisense probes are indicated in the panels: wild-type siblings (b–q), *unc45b* mutant embryos (b’–q’). All embryos are 72 h old. Anterior left, dorsal up. **r** Venn diagram of the genes upregulated in *ache*, *para* or *unc45b* mutants at 72 hpf. Note that in this case DESeq2 was used for the detection of up-regulated genes in order to increase the power of the differential expression analysis. **s** Log2 fold change of *unc45b*, *hsp90a*, *smyd1b* and *hspb1* obtained by RNAseq in 72-hpf *ache−/−*, *unc45b−/−* and *hsp90a−/−*. Asterisks above the bars indicate significant adjusted *p* values (*adjp*)

Hierarchical clustering and gene ontology (GO) analysis of the 1411 genes expressed differently between wild-type and *unc45b* mutant embryos revealed groups of co-regulated genes with shared gene ontologies (Fig. 2a; Additional file 4). Chaperones in general were strongly up-regulated in *unc45b* mutants (FDR < 10^−11^). In addition, we detected significant increases in the expression of genes involved in cardiovascular development (FDR < 10^−17^), muscle structure development (FDR < 10^−15^), cell proliferation (FDR < 10^−14^), in the response to hypoxia (FDR < 10^−12^) and related to angiogenesis (FDR < 10^−11^) (Additional file 4). Among the genes up-regulated in the mutant, 78 genes encode transcription factors (TFs) such as *mef2a*, *myod1*, *pax3a*, *rfx1*, *atf3* and *cepbg*, suggesting that the misfolded myosin phenotype activates complex myogenic regulatory networks. Besides up-regulated genes, we also detected a large number of genes whose expression levels were down-regulated (Fig. 2a). This group includes a significant proportion of genes involved in eye morphogenesis (FDR < 10^−13^ ) (Fig. 2a; Additional file 4).

To verify the results obtained by deep sequencing, we carried out RNA in situ hybridization on wild-type and *unc45b* mutant embryos. We selected 18 genes that were significantly up-regulated in our RNA-Seq dataset. The in situ hybridization results confirmed an increase in the expression of these genes in the *unc45b* mutants (Fig. 2b–q’). The three genes, initially indicated to be induced in *unc45b* mutant fish, *unc45b*, *hsp90a* and *smyd1b*, were also strongly up-regulated in our RNA-Seq dataset serving as a quality control for our sequencing data (Figure S1c–e in Additional file 2).

To exclude that the observed gene responses could be due to general cellular stress or caused indirectly by lack of heart beat and blood circulation, we analyzed the transcriptomes of *ache* as well as *hsp90a* mutants at 72 hfp (Additional file 1). We previously showed that *ache* mutants present skeletal muscle defects at 72 hpf [29], but have beating hearts and blood circulation [31]. *hsp90a* mutants have a skeletal myofibrilar phenotype identical to *unc45b−/−* but, in contrast to *unc45b−/−*, they do have a beating heart and normal blood circulation. We detected a total of 360 genes up-regulated more than 1.5 times (FDR < 0.05) in *ache*, 573 in *hsp90a*, and 1277 in *unc45b* mutants compared with wild-type sibling embryos. Comparison of the three transcriptomes revealed a set of 253 genes up-regulated in both *unc45b−/−* and *hsp90a−/−* but not in *ache−/−* mutants (Fig. 2r). GO analysis of this group revealed an enrichment of genes implicated in striated muscle development (*p* < 10^−2^), and response to unfolded protein accumulation (*p* < 10^−5^), including the chaperones *unc45b*, *hsp90a*, and *smyd1b* (Fig. 2s; Additional file 4). Notably, highly up-regulated genes in both *unc45b* and *hsp90a* mutants were generally not significantly regulated in *ache* mutants (i.e., *hspbp1*, *hsph1*, *hsc70*, and *hsp4a*; Figure S1f in Additional file 2). In contrast, genes down-regulated in *hsp90a−/−* were also down-regulated in *unc45b*−/−, but not in *ache−/−* (data not shown). Most of the genes up-regulated only in *ache* mutants were associated with the GO terms "apoptotic process" (*p* < 10^−4^) and "immune response" (*p* < 10^−3^) (Additional file 4). We found 59 genes up-regulated concomitantly in all three mutants, including the chaperone *hsp70l*. However, the level of upregulation of these genes in *ache−/−* is usually lower compared with those found in *hsp90a* and *unc45b* mutants as shown in Fig. 2s (i.e., *hsp70l* has a log_2_ fold change of 1.8 in *ache−/−*, compared with 6.2 in *hsp90a−/−* and 5.2 in *unc45b*−/−; Figure S1g in Additional file 2). There were 887 genes up-regulated only in the *unc45b* mutant (Fig. 2r). These genes include those involved in cardiac muscle development (*p* < 10^−4^), angiogenesis (*p* < 10^−5^), neural tube development (*p* < 10^−5^) as well as hypoxia (*p* < 10^−8^) (Additional file 4) and may thus be the consequence of heart failure in the mutant. Interestingly, cardiac developmental genes were induced similarly to the skeletal muscle developmental genes in the *unc45b* mutant, suggesting that the defects in muscle structure are compensated for by activation of developmental genes in both heart and skeletal musculature. Visual perception genes are specifically down-regulated in *unc45b−/−* (*p* < 10^−51^) (Additional file 4) and may be a result of the reduced size of the eyes observed in this mutant [1].

Together, these data demonstrate that lack of Hsp90a and Unc45b and the concomitant failure to fold myosin trigger a comprehensive and unique gene expression program in the mutant skeletal muscles that can be distinguished from other muscle stress-related changes.

### The kinetics of the transcriptional changes in *unc45b* mutants

**Fig. 3:**
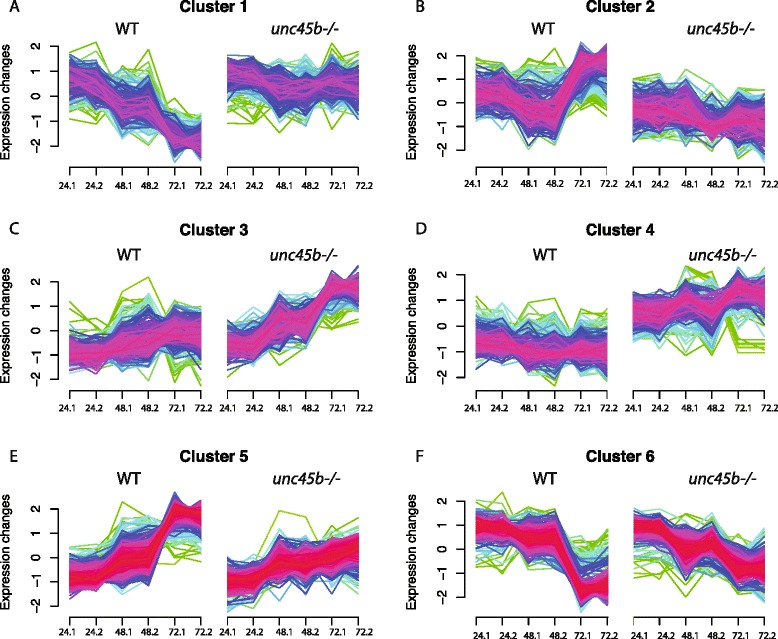
Distinct kinetics of gene responses in *unc45b* mutants. Soft clustering (k = 6) of mis-regulated genes in *unc45b−/−* embryos at 24 hpf, 48 hpf and 72 hpf in comparison with wild-type embryos. The expression levels were normalized and the y-axes indicate relative expression. The two independent measurements at each of the three time points are aligned along the x-axis of each graph. Genes with high membership after soft clustering are depicted with *red lines* and those with moderate membership with *green lines* and low membership with *blue lines*. Details on GO terms enriched in these clusters are provided in Additional file 6. **a** Cluster 1 represents genes that are down-regulated in wild-type (*WT*) siblings from 24–72 hpf. In contrast, these genes are maintained at a constant level in *unc45b* mutants over the same period. This cluster contains genes with a function in protein folding and maturation. **b** Cluster 2 genes are expressed at varying levels in 24-, 48- and 72-hpf wild-type siblings while they are maintained at relative constant expression levels in *unc45b* mutants. Genes in this cluster are associated with GO terms like cytoskeletal proteins in oligodendrocyte development and remodeling and cell adhesion. **c** Genes of cluster 3 rise moderately in their expression levels in wild-type siblings, while a much more pronounced increase is evident in the RNA samples isolated from *unc45b* mutants. Gene functions included in this cluster are blood vessel morphogenesis and skeletal muscle development. **d** Cluster 4 includes mRNAs of genes that are moderately down-regulated in wild-type siblings and increased in *unc45b* mutants over the three time points. These genes included genes with functions in protein folding. **e** Cluster 5 represents genes that are up-regulated in both wild-type and *unc45b* mutant embryos from 24–72 hpf but with a slightly lower slope in the mutant. Genes of this cluster are associated with a range of different GO categories, including cytoskeleton, intermediate filaments, and vesicle transport. **f** Cluster 6 includes genes that are down-regulated over the analyzed time period in both *unc45b* mutant and wild-type siblings even though the down-regulation in the mutant was less pronounced. GO terms of this cluster include developmental signaling, regulation of angiogenesis and neurogenesis

Given the complexity of the transcriptional response in *unc45b* mutants at 72 hpf, we asked whether gene groups differ in their kinetics of activation and repression. We sequenced the transcriptome of 24-hpf and 48-hpf *unc45b* mutant and wild-type siblings and compared the results with the 72-hpf RNA-Seq data. On a global scale, the maximum number of genes were activated at 72 hpf (Figure S1h–j in Additional file 2). By carrying out soft clustering (k = 6), we identified groups of genes that changed their level over the three time points in comparison to wild-type siblings in a characteristic manner (Fig. 3; Additional file 5). Clusters 1, 3 and 4 are composed of genes down-regulated over the course of development in wild-type embryos but remained highly expressed in *unc45b*^*−/−*^ embryos (Fig. 3a, c, d), including the genes *unc45b*, *hsp90a*, and *smyd1b*. Compared with wild-type embryos, the expression of these genes increased steadily in the *unc45b* mutant over the three time points (Fig. 3c; Figure S1e–g in Additional file 2). GO analysis showed that these clusters are enriched for protein folding, angiogenesis and skeletal muscle development (Additional file 6). Genes involved in cytoskeleton remodeling were strongly up-regulated in wild-type zebrafish at 72 hpf but remained at lower levels in *unc45* mutants (Fig. 3, clusters 2 and 5). Thus, taken together, lack of Unc45b activity induced complex patterns of gene responses that comprised not only chaperones but subsequently also other functions, including those involved in developmental processes.

### Skeletal muscle-specific expression and up-regulation of *unc45b* in myosin folding mutants is mediated by distinct 5’ regulatory elements

**Fig. 4:**
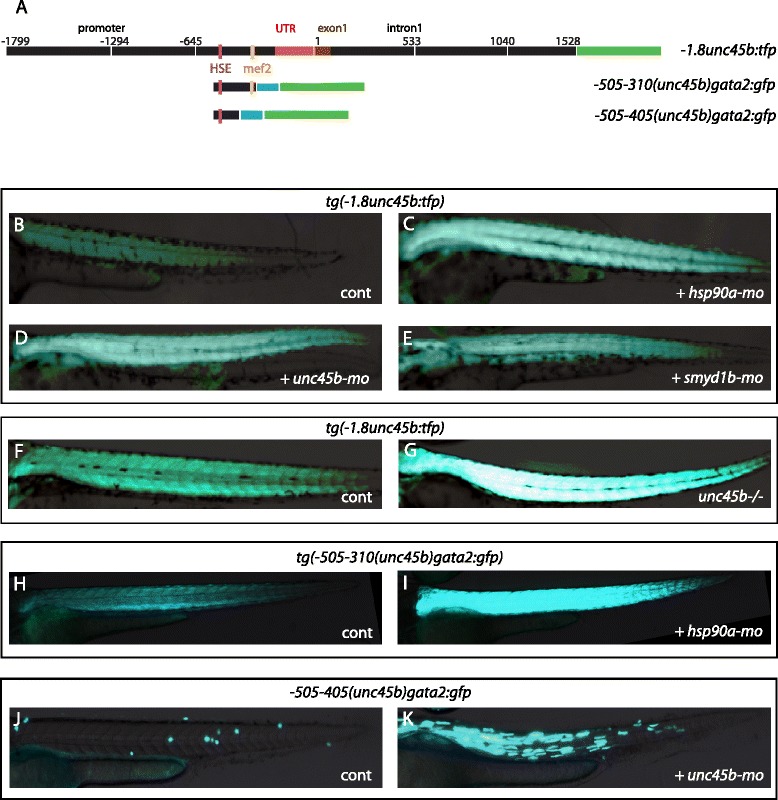
*unc45*-derived transgenes phenocopy the response to misfolded myosin. **a** Scheme representing the 3.3-kb sequence of the *unc45b* gene that recapitulates muscle-specific expression (*−1.8unc45b:tfp*) and its derivatives −*505/*−*310(unc45b)gata2:gfp* (Figure S3c in Additional file 8, construct 16) and −*505/*−*405(unc45b)gata2:gfp* (Figure S3i in Additional file 8, construct 31). All positions are indicted relative to the A of the ATG start codon (+1) of *unc45b. Red bars* represent the untranslated region (*UTR*). The first exon is indicated in *brown. Blue bars* represent the *gata2* minimal promoter. *Green bars* represent TFP or green fluorescent protein (GFP) reporter genes. The *pink vertical bar* indicates the heat shock element (*HSE*); the *yellow vertical bar* indicates the Mef2 binding motif. **b**–**e** Deficiency in myosin folding activates the −*1.8unc45b:tfp* construct. *Tg(−1.8unc45b:tfp)* embryos injected with either *hsp90a-mo* (**c**), *unc45b-mo* (**d**) or *smyd1b-mo* (**e**) show an increase of TFP compared with the uninjected control (**b**). **f**, **g** In comparison with transgenic wild-type sibling embryos (**f**), expression of *Tg(−1.8unc45b:tfp)* in *unc45b* mutant embryos (**g**) is elevated. This confirms the results from the morpholino knock-down experiments (**b**, **d**). **h, i** *Tg(−505/-310(unc45b)gata2:gfp)* embryos injected with *hsp90a-mo* (**i**) show an increase of GFP expression compared with the uninjected control (**h**). **j**, **k** Embryos injected with the construct −*505/*−*405(unc45b)gata2:gfp* show no GFP expression (**j**). However, co-injection with *unc45b-mo* triggers GFP expression in skeletal muscle (**k**). Thus, this transgene containing only 100 bp of the *unc45b* upstream region from −505 to −405 lost the basal muscle expression but retained the response to misfolded myosin. All embryos are 72 h old; anterior left, dorsal up

*unc45b* appears to be an early response gene to misfolded myosin; we observed up-regulation of its mRNA in the *unc45b* mutant already during somitogenesis stages. To decipher the mechanism leading to the induction of *unc45b* mRNA expression, we first tested whether 3.3 kb of the promoter region of the *unc45b* gene that recapitulates muscle-specific expression in transiently expressing zebrafish embryos [32] would mimic the transcriptional response to mutation of *unc45b*, *hsp90a* or *smyd1b*. This 3.3-kb fragment contains the *unc45b* promoter, including upstream and downstream regions (from −1799 to +1528 relative to the translation initiation site, including the first exon and first intron; Fig. 4a). All of the four independently generated stable transgenic lines (*tg(−1.8unc45b:tfp)*) drove expression of teal fluorescent protein (TFP) in skeletal and cardiac muscles (data not shown) that was indistinguishable from the pattern of expression of the endogenous *unc45b* gene [1]. *tg(−1.8unc45b:tfp)* transgenic embryos were injected with a morpholino directed against either *unc45b*, *hsp90a* or *smyd1b* mRNA to trigger the response to impaired myofibrilogenesis. In 100 % of the injected embryos (n = 200), we observed a strong up-regulation of the transgene (Fig. 4b–e; Figure S2a in Additional file 7). Similar results were obtained when we crossed the transgene into an *unc45b* or *hsp90a* homozygous mutant background (Fig. 4f, g; Figure S2 in Additional file 7; and data not shown). Thus, the 3.3-kb *unc45b* sequence contained in the transgene mediates the response in the myosin folding mutants.

To confirm this conclusion and to identify the regulatory sequence more precisely, we carried out a systematic deletion analysis and tested the capacity of the enhancers to drive expression when paired with the heterologous *gata2* promoter [33, 34] in either transient (Figure S3c, f, i in Additional file 8, constructs 12–15 and 17–42) or both transient and stable transgenic backgrounds (Figure S3c, d in Additional file 8, construct 16). A 195-bp fragment located between −505 and −310 bp (−*505/*−*310(unc45b)gata2:gfp*) mediated muscle expression and up-regulated green fluorescent protein (GFP) expression in *hsp90a* morphants in the four independent transgenic lines analyzed (Fig. 4h, i; Figure S2c in Additional file 7).

**Fig. 5:**
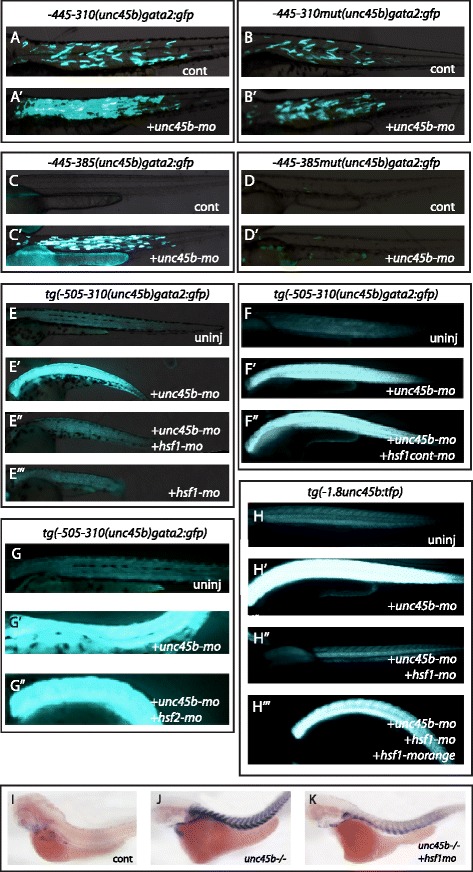
Hsf1 mediates the response to misfolded myosin. **a**, **a’** Co-injection of *unc45b-mo* with the construct −*445/*−*310(unc45b)gata2:gfp* (Figure S3f in Additional file 8, construct 25) leads to an increase of GFP expression (**a’**) compared with embryos injected with the plasmid alone (a). **b**, **b’** Co-injection of *unc45b-mo* with the construct −*445/*−*310mut(unc45b)gata2:gfp* in which the Hsf1 binding site was destroyed by four point mutations (Figure S3f in Additional file 8, construct 26) does not show an increase of GFP (**b’**) compared with embryos injected with the transgene alone (**b**). The basal muscle expression was unaffected by the point mutations. Thus, the Hsf1 recognition sequence is important for the transgene’s response in embryos with a myosin folding defect but not for basal expression in the muscle cells. **c, c’** The −*445/*−*385(unc45b)gata2:gfp* construct (Figure S3i in Additional file 8, construct 35) lacking the Mef2 binding site but containing the Hsf1 recognition sequence does not drive any GFP expression when injected into wild-type embryos (**c**). However, co-injection of *unc45b-mo* with this transgene triggers activation of GFP expression in muscle cells (**c’**). **d**, **d’** ***−*** *445/* ***−*** *385mut(unc45b)gata2:gfp* (Figure S3i in Additional file 8, construct 42) carries point mutations in the Hsf1 binding site in addition to a deletion of the Mef2 binding site. This construct does not show GFP expression when injected alone (**d**) or in combination with *unc45b-mo* (**d’**). Thus, in this construct both the basal expression in muscle cells and the misfolded myosin response are abolished. **e–e”’** Knock-down of Hsf1 (*hsf1-mo*) abolished the misfolded myosin response. Transgenic embryos stably expressing *Tg(−505/−310(unc45b)gata2:gfp)* were either not injected (**e)** (basal muscle expression) or injected with *unc45b morpholinos* (**e’**) (*unc45b-mo*, misfolded myosin induced expression) or double injected with morpholinos (**e”**) directed against *unc45b* and *hsf1* (*hsf1-mo*) or with *hsf1-mo* alone (**e”’**). Co-injection of *hsf1-mo* and *unc45b-mo* (**e”**) blocked the induction of the transgene as observed by injection of *unc45b* alone (**e’**). Injection of *hsf1-mo* alone (**e”’**) (compare with (**e”**) or (**e**)) did not alter basal muscle expression, demonstrating that Hsf1 is only required for the misfolded myosin response and not for basal muscle expression of the transgene. **f**–**f”** Injection of a *hsf1cont-mo* harboring five mismatches does not prevent the ability of −*505/*−*310(unc45b)gata2:gfp* to respond to the accumulation of unfolded myosin. *Tg(−505/−310 (unc45b)gata2:gfp)* was either not injected (**f**), or injected with the *unc45b-mo* (**f’**), or *unc45b-mo* and *hsf1cont-mo* together (**f”**). Expression of GFP reporter in double injected embryos (**f”**) is as high as in embryos injected with *unc45b-mo* alone (**f’**). **g**–**g”** Knock-down of Hsf2 (*hsf2-mo*) does not impair the response of *Tg(−505/−310(unc45b)gata2:gfp)* to misfolded myosin. *Tg(−505/-310(unc45b)gata2:gfp)* embryos were either not injected (**g**) or injected with *unc45b-mo* (**g’**), or *unc45b-mo* and *hsf2-mo* (**g”**). **h**–**h”’** Co-injection of the plasmid encoding Hsf1-mOrange fusion protein rescued the misfolded myosin response. *Tg(−1.8unc45b:tfp)* embryos were either not injected (**h**), or injected with *unc45b-mo* (**h’**) or with *unc45b-mo* and *hsf1-mo* (**h”**) or with *unc45b-mo*, *hsf1-mo* and *hsf1-morange* (**h”’**). The triple-injected embryos showed TFP reporter expression (**h”’**) comparable to that of embryos injected with *unc45b-mo* alone (**h’**). **i**–**k** Knock-down of Hsf1 (*hsf1-mo*) reduced the expression of *unc45b* mRNA from the endogenous gene in *unc45b* mutants. In situ hybridization with *unc45b* probe on either wild-type embryos (**i**), *unc45b* mutants (**j**), or *unc45b* mutants injected with *hsf1-mo* (**k**)*. unc45b* mutants were unequivocally identified by the lack of well-formed myofibrils and the total lack of motility. All embryos are 72 h old and are shown anterior left and dorsal up

By further deletion analysis (Figure S3f in Additional file 8, constructs 25–30), we mapped the region directing muscle-specific expression to the 55-bp region (−365 to −310) at the 3’ end of the 195-bp fragment (Figure S3f, h in Additional file 8, construct 30). Interestingly, this region harbors the binding site of the myogenic transcription activator Mef2 (Figure S4a in Additional file 9). Fragments depleted of the Mef2 recognition sequence (*−505/−405(unc45b)gata2:gfp*) did not show muscle-specific expression when expressed in a wild-type control but still mediated the response in *unc45b* or *hsp90a* morphants (Figs. 4j, k and 5c, c’; Figure S3i in Additional file 8, constructs 31–37; and data not shown). Thus, the response to loss of function of myosin folding genes and the muscle-specific basal expression can be separated from one another, pointing to independent and distinct mechanisms.

Next, we took advantage of the constructs that lack basal activity in the muscle but are activated by lack of myosin chaperones to re-address the issue of whether impaired myofibrillogensis triggers transgene activation. The −*505/*−*405(unc45b)gata2:gfp* construct was not induced in *titin* mutants *hel* and their wild-type siblings (Figure S4f in Additional file 9) and −*505/*−*310(unc45b)gata2:gfp* was not up-regulated compared with the expression in wild-type siblings (Figure S4g, h in Additional file 9). This supports our conclusion that impaired myofibrillogenesis is not an inducer of *unc45b* expression. Moreover, injection of low levels of *unc45b* morpholino into transgene-expressing embryos did not significantly impair myofibrillogenesis, as evident by the formation of striated fibrils, but it led to low level induction of the transgenes in the morphants (Figure S4c–e in Additional file 9). All together, these experiments exclude that lack of myofibrillogenesis per se triggers the up-regulation of *unc45b*, and implicates the accumulation of unfolded myosin as the key regulator of the response.

### Hsf1 is required for *unc45b* up-regulation in response to loss of function mutation in myosin folding genes

The region mediating the response to mutant myosin chaperones was compared with conserved regions in the *unc45b* genes of four other fish species. This comparison identified a conserved Hsf1 binding site called a heat shock element (HSE; Figure S4a, b in Additional file 9; Figure S3j in Additional file 8). When this Hsf1 site was mutated by introducing point mutations in the recognition sequence (compare −*445/*−*310(unc45b)gata2:gfp* with −*445/*−*310mut(unc45b)gata2:gfp*; Fig. 5a, b’; Figure S3f in Additional file 8, constructs 25 and 26; Figure S3i in Additional file 8, constructs 38, 41, and 42) or deleted (Figure S3f in Additional file 8, constructs 27–30; Figure S3g, i in Additional file 8, constructs 39–40) the transgenes did not elicit an increase of reporter expression in *unc45b* morphants. Thus, the Hsf1 binding site is required for the response to misfolded myosin.

We next tested whether the TF Hsf1 is indeed necessary to mediate the up-regulation of *unc45b* in response to misfolded myosin. We knocked-down the translation of *hsf1* by injecting a morpholino directed against *hsf1* mRNA (*hsf1-mo*). To induce the response to myosin misfolding mutants, we co-injected morpholinos either directed against *unc45b*, *hsp90a* or *smyd1b* mRNA into the stable line *tg(−505/-310(unc45)gata2:gfp).* We compared GFP expression in uninjected embryos with that in embryos injected with *unc45b-mo* alone. Co-injection of *hsf1-mo* with *unc45b-mo* decreased the GFP signal to the basal level comparable to that observed in an uninduced line (Fig. 5e–e”’; Figure S2d in Additional file 7; 100 % injected embryos, n = 40). A mismatched *hsf1* morpholino control (*hsf1cont-mo*) did not change the GFP expression level (Fig. 5f–f”; Figure S2f in Additional file 7). To verify that the lack of Hsf1 is indeed responsible for the decreased GFP expression, we cloned an Hsf1-mOrange fusion protein to rescue the *hsf1* knock-down. Injection of *hsf1-morange* together with *unc45b-mo* and *hsf1-mo* into the *tg(−1.8unc45b:tfp)* line restored the activity of the reporter construct to levels seen in *unc45b*-*mo* injected transgenic controls (Fig. 5h–h”’). As further specificity control, we used a transient Crispr/Cas9 knock-out approach [35] to remove Hsf1 and obtained similar results (Figure S5a in Additional file 10). Altogether, these data indicate that the observed suppression of the response in myosin folding mutants in Hsf1 loss-of-function experiments was specific.

Since both Hsf1 and Hsf2 were implicated in control of the cellular stress response [36, 37], we tested if Hsf2 could also play a role in the misfolded myosin response. Injection of *hsf2-mo* did not block the induction of GFP expression in the *tg(−505/-310(unc45)gata2:gfp)* line in response to knock-down of *unc45b*, *hsp90* or *smyd1b* (Fig. 5g–g”; Figure S2e in Additional file 7; and data not shown). Thus, Hsf2 does not mediate the response observed in myofibers with misfolded myosin.

Next, we asked whether Hsf1 is required for basal expression of *unc45b* in muscle cells. Injection of *hsf1-mo* into the *tg(−505/-310(unc45)gata2:gfp)* or *tg(−1.8unc45b:tfp)* line did not result in a decrease of GFP/TFP expression (Fig. 5e, e”’, and data not shown). Overexpression of Hsf1-mOrange in the *tg(−1.8unc45b:tfp)* or *tg(−505/−310(unc45b)gata2:gfp)* lines did not increase GFP/TFP expression under normal homeostasis, or in the myosin folding mutants (Figure S5b, b’ in Additional file 10, and data not shown). This indicates that Hsf1 is not involved in the maintenance of the *unc45b* basal level of expression.

We also investigated whether the endogenous *unc45b* gene would be impaired in its response in an *unc45b* mutant background by knock-down of Hsf1. To this end, we injected the *hsf1-mo* into *unc45b* mutants and analyzed the level of the endogenous *unc45b* mRNA by in situ hybridization with antisense probe [1]. Knock-down of Hsf1 reduced the induced levels of *unc45b* mRNA (Fig. 5i–k). In summary, we conclude that Hsf1 is necessary to trigger the activation of *unc45b* in myosin folding mutants.

Since Hsf1 is a heat shock factor, we tested whether *unc45b* transgenes could also be activated by heat shock. Expression from the *Tg(−503-510(unc45b)gata2:gfp)* or *Tg(−1.8unc45b:tfp)* line was increased after incubation of embryos over night at 37 °C in comparison with controls kept at 28 °C (Figure S5c–h’ in Additional file 10). However, we did not observe a temperature-dependent response of the transgenes when embryos were incubated for periods shorter than 12 h at the elevated temperature.

### Hsf1 binding sites are enriched in the upstream regions of genes regulated in response to loss-of-function of *unc45b*

We next assessed whether Hsf1 binding sites are enriched in the promoter regions of the genes that are significantly up- or down-regulated in *unc45b* mutant larvae. We selected 1000-bp promoter sequences of the genes of each of the six clusters (Fig. 3), thereby discriminating gene groups by their characteristic expression kinetics in wild-type and *unc45b* mutant embryos. A highly significant enrichment of Hsf1 binding sites was detected in the promoters of genes belonging to cluster 4 (*p* < 10^−197^; Table 1; Figure S6a in Additional file 11). These genes are involved in cellular stress response (*hsp70l*, *hspa4a*, *hsp90aa1.1*), clearance of dysfunctional proteins via ubiquitination and chaperone-assisted selective autophagy (*bag3*, *ubc*, *usp2a*, *uchl1*) [38] (Additional file 12). We also found Hsf1 binding sites in other genes, such as *smyd1b*, the cell cycle regulator *mcm5*, *smarca2* (*SWI/SNF-related matrix-associated actin-dependent regulator of chromatin*) and the TFs *mlf1*, *nfe2l1a* and *tfe3a*. This suggests that, in addition to the direct activation of genes with a function in protein folding and turnover, other biological processes can also be directly regulated by Hsf1. A marginal enrichment of Hsf1 binding sites was found in the 1000-bp promoter sequence of genes belonging to cluster 1 (*p* < 10^−5^; Table 1). These genes have expression kinetics similar to cluster 4 (Fig. 3), and are probably part of the same network of regulation during the response of the transcriptome to misfolded myosin. In line with this assumption, we detect in this group Hsf1 binding sites in the promoters of *unc45b* and the gene encoding sarcalumenin, a calcium-binding protein found in the sarcoplasmic reticulum of striated muscle (Additional file 12). Hsf1 binding sites were also enriched in the set of genes up-regulated in *unc45b−/−* and *hsp90a−/−* mutants (z-score > 33; Additional file 13). None of the genes up-regulated in *ache* mutants showed enrichment in HSEs.

**Table 1:** Summary of the Hsf1 binding sites detected in the six gene groups obtained by fuzzy mean clustering

Cluster	Total genes	Hsf1 containing genes	*P* value
1	160	17	1.1E-05^a^
2	183	16	2.1E-02
3	168	10	2.2E-04
4	178	35	9.8E-198^b^
5	428	30	3.2E-01
6	204	12	4.6E-02

The total number of genes in each cluster is indicated, as well as the *p* value obtained for the enrichment of Hsf1 binding site and the total number of target genes for which an Hsf1 binding site is detected (*p* value < 10^−20^)

^a^Marginal Hsf1 binding site enrichment

^b^Significant Hsf1 binding site enrichment

To assess whether aspects of the Hsf1 response are conserved, we searched human orthologs for the presence of HSEs. We found conserved enrichment of HSEs in the promoters of human orthologs of cluster 4 genes, including *HSP90*, *HSP70*, *HSP8*, *BAG3*, *UBC* and *UNC45B* (Figure S6b in Additional file 11; Figure S6c in Additional file 11). HSF1 binding data do not exist for human skeletal muscle. We thus queried the only available human HSF1 ChIP-Seq data set, derived from the hepatocellular carcinoma cell line HepG2 [39]. Physical binding of HSF1 was detected in the promoters of 10 out of the 19 up-regulated TFs with predicted HSEs (Table 2): *atf5b*, *nfe2l1*, *CREB3L3*, *stat5.1*, *tcf3*, *znf800b*, *hoxc1a*, *klf15*, *neurog1* and *tefa*. Taken together, this suggests that at least some components of the Hsf1 response are conserved in the human genome.

**Table 2:** Detection of HSE in TFs up-regulated during response of the genome to misfolded myosin

Name	Cluster	P_adj_	Hsf1 sequence	Position	24-hpf expression	Human ChIP peak
CREB3L3	1	4.9E-15	TCTCCAGAAACATCC	1973–1987 (intron)	Not restricted	Yes
myod1	1	1.5E-04	GTTCTGGAACATTAC	1658–1672 (exon1)	Somite	No
mxtx1	1	1.6E-05	TTTCAAGAAATTTCT	574–588 (promoter)	Not restricted	No ortholog
zgc:113263	1	4.9E-05	CTTCCTGAAGTTTCG	659–673 (promoter)	Not restricted	No ortholog
atf5b	4	1.4E-04	TTTCTAGAGACTTCC	1295–1309 (intron)	NA	Yes
nfe2l1	4	2.3E-05	TTTCCAGAATATTTT	876–890 (promoter)	Not restricted	Yes
AL929286.1	4	8.5E-17	TGTCCAGCACCTTCT	1278–1292 (exon1)	NA	No ortholog
znf395a	4	5.1E-34	TTTCTAGAACATTAT	178–192 (promoter)	Not restricted	No
			TTTCTAAAACATTCC	1880–1894 (intron)		
tfe3a	4	3.1E-06	TTTGCAGAATCTTCC	250–264 (promoter)	Blood, YSL, lens	No
stat5.1	6	1.4E-02	ATTCCGGAAGCTTCT	341–355 (promoter)	Somite	Yes
tcf3	6	4.3E-03	TCTCTGGCAAATTCT	328–342 (promoter)	Not restricted	Yes
hoxc1a	6	1.5E-02	CTTTCAGAACTTTCT	1359–1373 (exon1)	Spinal cord	Yes
klf15	6	7.3E-03	TTTCCAGAATTTTTT	330–344 (promoter)	Somite	Yes
neurog1	6	2.2E-02	TTTCTGGCGTATTCC	1180–1194 (intron)	Nervous system	Yes
vox	6	4.5E-02	TTTCTGAAATATTCT	1550–1564 (intron)	Not restricted	No
sim1a	6	3.6E-02	TTTCTTGAAGTCTCT	957–971 (promoter)	Diencephalon	No
			TATCCAGAAGGATCT	986–1000 (promoter)		
znf800b	NA	5.1E-03	TTTCCAGAAGAGTCG	638–652 (promoter)	NA	Yes
tefa	NA	8.2E-04	CTTCCAGAAGATTCG	1289–1303 (exon2)	Not restricted	Yes
znfl2a	NA	9.9E-02	TTTCTAGCTCTTTCT	827–841 (promoter)	Blood	No ortholog

The 19 TFs up-regulated during the misfolded myosin response with identified Hsf1 binding sites. Position of the potential Hsf1 binding site (*p* values are indicated) relative to the transcriptional start site is indicated with a reference to promoter, exonic and intronic location in parentheses. Data from RNAseq (clusters obtained by fuzzy mean clustering), expression patterns observed at 24 hpf [43], as well as HSF1 occupancy from human orthologs obtained from ChIP are indicated, if available. *NA* not available

**Fig. 6:**
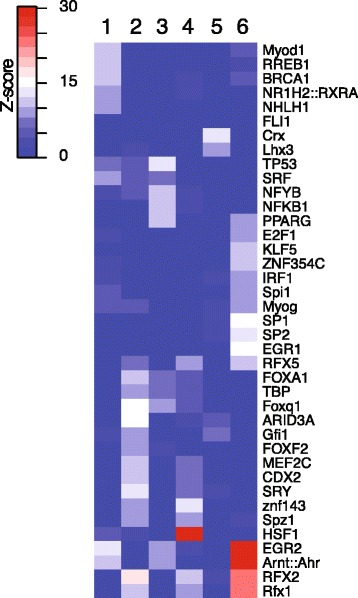
In silico mapping of Hsf1 binding sites. Z-scores of TF binding sites enriched in 1-kb promoter sequences for each cluster group (clusters 1–6) obtained by fuzzy mean clustering. The scale bar indicates the significant enrichment of specific TF binding sites in a given gene group (significant Z-scores on scale bare from *white* to *red*)

No enrichment of Hsf1 binding sites was scored in the promoter region of genes of clusters 2, 3, 5 and 6 (Table 1). But other TF binding sites are enriched (Fig. 6). For example, clusters 2 and 6 are enriched for putative RFx1 and RFx2 binding sites (*p* < 10^−20^). Genes in cluster 3 are associated with the GO term “programmed cell death” (FDR < 10^−6^) and indeed these genes are enriched for TP53 binding motifs (*p* < 10^−36^), providing a positive control for the sensitivity of our analysis [40]. Cluster 5 is associated with visual perception (FDR < 10^−5^) and, in agreement, we found putative binding sites in these genes for the homeobox TF genes *crx* and *lhx2* (*p* < 10^−18^), which are both involved in retina development [41, 42]. Promoters of genes belonging to cluster 1 are enriched for other TF binding motifs, in particular Myod1 and Nhlh1 binding motifs (*p* < 10^−20^).

The genes of clusters 2, 3, 5 and 6 may be regulated indirectly via activation of TF genes by Hsf1. We thus examined specifically the promoter and first intron of the 88 TF genes up-regulated in *unc45b* mutants (FDR < 0.1, fold change > 1.5) for HSEs. We found putative Hsf1 binding sites in 19 TF genes. These include *myod1*, *sta5.1*, and *klf15*, expressed specifically in the developing somites, as well as seven TFs such as *mxtx1*, *nfe2l1* and *CREB3L3* expressed ubiquitously, and *tfe3a*, which is expressed in blood precursors in 24-hpf embryos [43] (Table 2). These data suggest that the immediate response to Hsf1 could be further amplified by induction of downstream TFs.

## Discussion

Loss of function in zebrafish *unc45b* and *hsp90a* genes leads to failure to assemble myosin into ordered myofibrils. Here, we show that the response of the transcriptome to loss-of-function of *unc45b* and *hsp90a* involves increased expression of genes primarily implicated in protein folding and muscle development, and identify Hsf1 as the key regulator of the expression of genes involved in myosin folding. We detected a Hsf1 binding motif in the *unc45b* promoter and show that this site is essential for *unc45b* up-regulation in muscle fibers with accumulated misfolded myosin. Deep sequencing of the transcriptome indicates that the response is shared by *unc45b* and *hsp90a* mutants with defective myosin folding and is significantly different from the *ache* mutant that has defective sarcomere assembly.

### Regulation of *unc45b* expression

*unc45b* is expressed in the skeletal and cardiac muscle of the developing wild-type embryo. Lower levels of *unc45b* expression were recently reported in the lens and retina of wild-type embryos [22]. The levels of *unc45b* appear to be crucial for myofibrillogenesis. The efficient folding of myosins as major structural components of the myofibril requires appropriate levels of auxiliary chaperones (Unc45b, Hsp90a and other proteins). The fact that the expression of the myosin chaperone genes is induced in the mutant backgrounds suggests that there is a transcriptional mechanism to adapt the levels of myosin folding proteins.

Our mutational analysis of the *unc45b* gene provided evidence for two *cis*-regulatory mechanisms. The basal muscle-specific expression of the *unc45b* reporter transgene is driven by a region containing a recognition sequence of the TF Mef2 [44–46] and deletion of this region leads to loss of basal expression in the muscle. Expression could, however, still be induced in response to misfolded myosin in morphants/mutants of *unc45b*, *hsp90a* or *smyd1b*. Thus, basal muscle expression can mechanistically be uncoupled from the response to impaired myosin folding genes.

A region different from the one mediating basal expression of the *unc45b* 5’ regulatory region is responsible for the response to defective myosin folding. This region contains sequence homologous to an HSE [47, 48]. Indeed, when we mutated the HSE by introducing four point mutations, the transcriptional response was abolished. Zebrafish expresses two Hsfs, Hsf1 and Hsf2 [48]. The response triggered in myofibers with misfolded myosin was abolished by knock-down of *hsf1* but not *hsf2*. This implies non-redundant functions of the two Hsf factors and a highly specific role of Hsf1 in mediating the response.

**Fig. 7:**
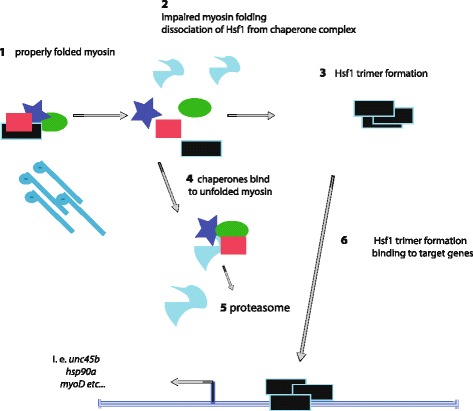
Model of the response to misfolded myosin. Under normal conditions chaperones such as Hsp40, Hsp90 and Hsp70 sequester Hsf1 monomers (1) [83] in the cytoplasm. Upon accumulation of unfolded myosin in *unc45b*, *smyd1b* and *hsp90a* mutants, the chaperones bind to the misfolded myosin and release Hsf1 (2, 4). Remaining unfolded myosin is sent to the proteasome (5). Hsf1 monomers then form active homotrimers (3) which bind to HSEs in target genes (6), such as those of *unc45b*, *hsp90a* and *myod.* More than 1400 genes activated in the misfolded myosin response include those encoding TFs that indirectly activate downstream gene programs and thereby further diversify the response of the transcriptome

Hsf1 is regarded as the major mediator of cell stress signals [47, 49–53]. Based on previous studies on the regulation of Hsf1 [49, 54], we propose the following hypothetical model (Fig. 7). Hsf1 is kept in an inactive complex either with heat shock proteins such as Hsp70 and Hsp90 [55, 56] or by shuttling between the nucleus and the cytoplasm [55]. In the absence of Unc45b (or Hsp90a or Smyd1b), misfolded myosin accumulates and interacts with the heat shock protein partners of Hsf1, thereby releasing the Hsf1 monomer. Alternatively, nuclear retention of Hsf1 could be increased [55] and Hsf1 trimers interact with the HSE of the *unc45b* gene, thereby activating its expression [57, 58]. Accordingly, the initial trigger would be provided by the accumulating misfolded myosin in the mutants (see below for a discussion). We cannot, however, exclude that other cues activate Hsf1. In particular, the precise mechanisms of activation of Hsf1 by cellular stress is still under dispute (for review see [49]).

One prediction of the model is that we could also induce *unc45b* by heat shock that would trigger DNA binding of Hsf1 as previously indicated [59]. We indeed observed an increase in *unc45b* mRNA after 12 hours of induction at 37 °C. However, the kinetics of this induction was much slower than the normal response to heat shock that occurs within 30–60 minutes [60, 61]. This suggests that the response appears to involve modulators that take the state of myosin folding and sarcomeric assembly in elevated temperature into account. Moreover, *smyd1b* expression, although up-regulated in response to the misfolded myosin condition, does not respond to heat or cold shock [15]. In this context it may be of importance that Hsf1 is subject to phosphorylation, acetylation and sumoylation, which appear to regulate various aspects of its function (for review see [49]). Also, mere overexpression of Hsf1 in wild-type zebrafish muscle is not sufficient to activate *unc45b* expression above that of controls. This supports the notion that additional factors are required for activation of the transcriptome in response to impaired myosin folding.

Whereas limited expression of myosin chaperones impairs myosin folding, excessive Unc45b protein is also detrimental to the function of the myofiber [25]. In *Caenorhabditis elegans*, the levels of Unc45b protein are regulated by interaction with the ubiquitinilation/degradation complex CDC-48–UFD-2–CHN-1 [26, 27]. Mutations in the human homolog of CDC-48, named p97, result in the elevation of Unc45b protein levels and cause hereditary inclusion body myopathy in humans [27]. Forced overexpression of *unc45b* is also detrimental to myofiber structure in zebrafish embryos [25]. In this context, it may also be of importance that the components of the Unc45b–Hsp90a–Smyd1b myosin folding complex do not remain associated with the folded myosin in the A-band but rather accumulate either at the Z-line (Hsp90a, Unc45b) [12] or the M-line (Smyd1b) [13] in the mature fiber. This suggests that the levels of the available myosin chaperones are regulated by association with these myofibrillar structures. The contractile apparatus is subject to rapid remodeling dependent on the nutritional status and health condition of the animal and, as expected, Hsp90 and Unc45b were thus found to be upregulated after feeding of malnourished zebrafish [62]. Taken together, these data suggest that Unc45b and interacting chaperones are regulated at multiple levels from gene transcription and protein stability to subcellular location to achieve optimal levels of the chaperones under various physiological conditions and to prevent pathological proteotoxic effects in cells.

### Misfolded myosin as a possible inducer of the transcriptome response

The *unc45b*, *hsp90a* and *smyd1b* mutants are characterized by the same myofibrillar defects [1, 9, 13]. Moreover, the mutants accumulate aberrant myosin deposits in skeletal myofibrils that are not arranged into thick filaments as in wild-type embryos. The three genes interact [11, 13, 15, 63] and for two of them a direct function as myosin chaperones has been well documented from invertebrates to vertebrates [11, 17]. While *hsp90a* is expressed only in the skeletal musculature, the other two genes, *unc45b* and *smyd1b*, are also expressed in the heart [1, 9, 13]. Thus, a common denominator of all three genes is a role as chaperones or co-chaperones for myosin folding in the skeletal musculature of the zebrafish embryo. *Unc45b* and *smyd1b* have these functions additionally in cardiac muscle [1, 9, 13]. Loss of function of each of the three genes causes up-regulation of the mutant gene’s own expression as well as that of the two partner genes. We showed here that this response entails a large number of additional genes.

A crucial question is what is the signal that triggers this response of the transcriptome? It is well documented that in Duchenne muscular dystrophy and corresponding animal models, expression of the mutated dystrophin gene is down-regulated [64, 65]. Thus, the transcriptional response of the myosin chaperone genes in myosin folding myopathy is in sharp contrast to the dystrophin response in muscular dystrophy. From careful comparison of various zebrafish motility mutants with *unc45b*, *hsp90a* and *smyd1b* as key probes, we excluded that paralysis or cellular stress of the myofiber are causes of the response. This conclusion was further supported by transcriptome-wide analysis of the *ache* mutants that show lower regulation of other stress proteins like *hspb11* [66] and genes belonging to apoptosis and immune response ontologies. Analysis of *titin* morphants and the *titin* mutant *hel* showed that failure of myofibrillogenesis is also not the trigger of increased *unc45b*, *hsp90a* and *smyd1b* expression. Since *titin* mutants express lower levels of myosin proteins, we believe that the lower levels of myosin seen in *unc45b* and *smyd1b* mutants can also be excluded as a potential trigger for the observed transcriptional response. This conclusion was independently confirmed by myosin Myhc4 knock-down in wild-type embryos. A remaining candidate for inducing the transcriptome response and up-regulation of myosin folding-related genes is misfolded myosin itself that accumulates in the cytoplasm in the three chaperone mutants. This model is appealing as it provides a direct link to Hsf1, the transcriptional mediator of *unc45b* induction: Hsf1 is known to be activated by misfolded proteins [49]. Furthermore, expression of a mutant myosin with several missense mutations disrupting the secondary structure of the head domain lead to activation of *unc45b* transgenes in the yolk cell. We thus propose to name this response of the transcriptome the "misfolded myosin response" or MMR.

#### The response of the genome to misfolded myosin is complex

The response of the transcriptome to *unc45b* deficiency was surprisingly large, with more than 1400 genes significantly altered in their expression at 72 hours. Approximately 900 genes were up-regulated and about 500 genes were down-regulated in the mutants at 72 hpf relative to wild-type siblings. Recently, the chaperone/co-chaperone interaction network has been elucidated in human cells, indicating a highly complex system to facilitate specific folding of diverse client proteins [4]. The transcriptome response to misfolded myosin appears to trigger the activation of both of the main nodes in the chaperoning network — Hsp90 and Hsp70 complexes — and can thereby influence a large number of client proteins. The regulated genes include a large number of chaperones but also genes involved in muscle structure development, cardiovascular development and cell proliferation. Thus, the mutant embryo appears to compensate for the *unc45b* deficiency by a genome-wide expression profile change that includes genes involved in processes other than protein folding. Increased expression of genes playing a role in angiogenesis and hypoxia may, for instance, be a reflection of the cardiac defect in *unc45b* mutants. To pinpoint the transcriptional response that possibly arises from misfolded myosin accumulation, we took a comparative approach using several zebrafish mutants. The heart muscles of *hsp90a* mutant fish are unaffected and share with the *unc45b* mutants only defective skeletal muscle myosin folding. *Ache* mutants suffer from skeletal muscle paralysis that does not, however, affect myosin folding. By comparative analysis of the transcriptomes of *unc45b*, *hsp90a* and *ache* mutants we could identify the gene expression signature that is characteristic of skeletal muscle defects arising from lack of functional myosin folding machinery and is distinct from changes that are triggered by lack of heartbeat in the *unc45b* mutant or by paralysis in the *ache* mutant.

Among the up-regulated genes in the *unc45b* mutant we found 88 genes that belong to the GO group “transcription regulator”, including *mef2a*, *myod1* and *pax3a*, known to be regulators of muscle-specific genes. Thus, the response to *unc45b* deficiency may not entirely be mediated by Hsf1 and most likely includes a further amplification of the transcriptional response by these downstream TFs. Although the skeletal myosins are the most abundant myosins in the myofiber, we cannot exclude that misfolding of the non-muscle myosins, such as the myosins implicated in the formation of costameres mediating fibril attachment [21, 23], co-determines the types of genes induced in the *unc45b* mutant. Interestingly, a significant number of genes involved in visual perception (such as Alpha crystallin A [67], CNGA3 [68], and RLBP1 [69]) are down-regulated in the *unc45b* mutant. This is correlated with smaller eyes in the *unc45b* mutant and may indicate an as yet ill-defined function of Unc45b in the eye. A low level of *unc45b* expression was recently detected in the lens as well as in the ganglion cell layer. *unc45b* mutants develop cataracts [22]. A function of *unc45b* in the retina, which may account for the lower abundance of gene transcripts involved in visual perception in *unc45b* mutants, has not been described.

Transcriptome analysis during zebrafish embryonic development enabled us to identify clusters of genes that change expression in the *unc45b* mutant in similar patterns. For example, we could identify genes that are maintained at high levels in the *unc45b* mutant fish from the early somitogenesis stage and another cluster of genes that are up-regulated only in the later embryonic stage in comparison to wild-type embryos. Interestingly, genes encoding chaperones are distributed among early and late responders, indicating that protein folding machinery in the myofiber is dynamically altered by progressive stress that is inflicted by absent Unc45b and concomitant accumulation of unfolded myosin.

Genes that are upregulated late in larval stages of the *unc45b* mutant (cluster 4) show an enrichment in Hsf1 binding motifs, suggesting that not only *unc45b* but also many other genes of the misfolded myosin response are regulated by Hsf1. Clearly, there are biases in the in silico analysis, such as the restriction of the study to the 1-kb upstream sequence, which may preclude the detection of HSEs in further genes. However, despite this fact, it is obvious that other clusters show either a lower (cluster 1) or no enrichment (clusters 2, 3, 5, and 6) of HSEs in the immediate promoter upstream region. This indicates that most likely not all genes which are part of the transcriptome response in *unc45b* mutants are direct targets of Hsf1. In agreement with such a notion, we detected 19 HSE-containing TF genes (including the muscle differentiation factor myod [70]) among the 88 TF genes. Thus, the response may be amplified via Hsf1-medated activation of downstream TF genes. This may explain the regulation of genes in the clusters with low or no significant enrichment of HSEs (clusters 1, 2, 3, 5, and 6) and is also a likely cause of the different kinetics of the six clusters over developmental time.

## Conclusion

Skeletal muscle cells have a highly specialized transcriptional feedback mechanism that links the activity of myosin chaperone proteins with expression of a large number of downstream genes. One key regulator of this response is Hsf1. The expression of the chaperones is induced for refolding or removal of misfolded protein, thereby preventing proteotoxic effects in the myofiber. The response to impaired folding of myosins also entails changes in the transcriptional status of many other genes with different functions. Expression of developmental genes is elevated, including TFs that control muscle differentiation, suggesting that myosin folding is coupled to muscle differentiation. The observed complexity of the response to misfolded myosin accumulation is most likely a reflection of the plasticity of myofibrils during the life of an animal, serving not only as motors for body movement but also as a store for amino acids and energy.

## Material and methods

### Fish stock

Fish were bred and raised as previously described [71]. The following mutant alleles were used: *unc45b*^*sb60*^ [1], *sop*^*fixe*^ [28], *hsp90aa1.1*^*tu44c/tu44c*^, [9] *smyd1b*^*zf340/zf340*^ [13], *ache*^*sb55*^ [29], and *herzschlag* (*hel*^*tg287*^) [72].

### Cloning

*unc45b* regulatory sequences were cloned into the vector described in [32] corresponding to the modified pT2KXIGΔin [73], upstream of the monomeric Teal fluorescent protein 1 (mTFP1). For cloning *gata2* based reporter constructs, the *unc45b* fragments were inserted in front of the *gata2* promoter [33] driving expression of *gfp* by Gateway cloning [34]. *unc45b* upstream sequences were amplified and cloned following standard procedures. Details are available upon request.

A full-length cDNA encoding zebrafish *hsf1* (IRBOp991H0894D, Imagene) was amplified (primers available upon request). The resulting PCR product was cloned into a vector containing 3.3-kb *unc45b* regulatory sequence upstream and in-frame of the monomeric orange fluorescent protein 1 (mOrange1) [74]. TF binding sites were identified with Genomatix.

### Western blotting

Protein was extracted by homogenization of deyolked embryos and separated by 10 % SDS-PAGE, transferred onto a nitrocellulose filter, and incubated with different primary antibodies (1:100 F59 DSHB and 1:100 γ-tubulin, Sigma]) for one night at 4 °C. After washing three times, a secondary antibody (goat anti-mouse Alexa Fluor 680, Invitrogen) was applied for 1 h at room temperature. Blots were visualized using an infrared imaging system (Odyssey; LI-COR Biosciences).

### Microinjection

Microinjection was carried out as described [75], briefly, 1.5–2 nl of injection solution containing 20 ng/μl reporter plasmid DNA and 15 ng/μl Tol2 transposase mRNA, supplemented with 0.1 % phenol red (injection marker), was injected into zebrafish eggs using a FemtoJet microinjector (Eppendorf).

Morpholinos (Genetools LLC, Oregon) were injected as follows: 0.3 mM *unc45b-mo* (CCAATTTCTCCCATCGTCATTGAAG) [1]; 0.1 mM *hsp90a-mo* (TCGAG TGGTTTATTCTGAGAGTTTC) [9]; 0.3 mM *smyd1b-mo* (AAAAACTTCCAC AAACTCCATTCTG) [13]; 0.3 mM *hsf1-mo* (CACGGAGAGTTTAGT GATGATTTCT) [51]; 0.3 mM *hsf1cont-mo* (CACGCACAGTTTACTGATCAT TTGT); 0.3 mM *hsf2-Mo* (GACGTTCGA GCTGTGTTTCATTTTG) [51]; and 0.4 mM *titin-Mo* (GTGGAAGACCGG TAAGATTACATCT) [77, 76].

### In situ hybridization and imaging

Whole-mount in situ hybridization was performed as described [77]. Bound antisense probe was revealed with anti-DIG alkaline phosphatase (Roche). The probes for *mef2d*, *myoD*, *atf3*, *vgll2b*, *SRFl*, *klhl31*, *kbtbd10b*, *kelch-like*, *trim55b*, *mef2a*, *nr4a1*, *smyd1a*, *crip2*, *fhl2a5*, *sarcosin6* and *trim55a* were obtained from [43] and for *unc45b*, *smyd1b*, and *hsp90a* from [1, 13].

All images were taken with a Leica microscope (MZ16F) and Leica camera (DFC320). GFP and TPF intensity was measured with ImageJ (http://imagej.nih.gov/ij/).

### RNA-Seq analysis

Pools of 20–50 zebrafish embryos with wild-type or *unc45b* mutant phenotype were collected at 24, 48 and 72 hpf from two independent clutches. Total RNA extraction was performed with Trizol (Invitrogen) following the manufacturer’s protocol. Extracted total RNA samples were tested on RNA nanochips (Bioanalyser 2100, Agilent) and showed no sign of degradation (RNA index number > 9). Sequencing libraries were generated from 1 μg of RNA samples with the TruSeq mRNA kit v.2 (Illumina). Size and concentration of sequencing libraries were determined with DNA-chip (Bioanalyser 2100, Agilent) and the concentrations adjusted to 7 pM. Multiplexed samples were loaded on a total number of three sequencing lanes. Paired end reads (2 × 50 nucleotides) were obtained on a Hiseq1000 using SBS v3 kits (Illumina).

Cluster detection and base calling were performed using RTA v.1.13 and quality of reads assessed with CASAVA v.1.8.1 (Illumina). The sequencing resulted in 302 million pairs of 50-nucleotide-long reads with a mean Phred quality score > 35 (Additional file 1). The reads were mapped against the zebrafish genome (Zv9) using TopHat version 1.4.1 [78] with the options --butterfly-search --coverage-search --microexon-search -a 5 -p 5 --library-type fr-unstranded and using known exon junctions (Ensembl release 75). The mean distance and standard deviation between read pairs were obtained from CASAVA. Gene expression was determined with HTSeq version 0.5.3p3 [79] by counting for each gene the number of reads that overlapped with the annotation location obtained from Ensembl release 75. Differential expression was calculated using the *R* package DESeq [79]. Genes with 1.5 fold change (increase or decrease) and adjusted *p* value (FDR) less than 0.05 were considered as differentially expressed. Hierarchical clustering was performed in *R* with the *gplots* package on a set of selected genes differentially expressed in at least one condition with Pearson's correlation and the complete-linkage method, using variance stabilized expression data. Fuzzy clustering was performed on a set of 1825 genes misregulated in at least one condition (FDR < 0.05) using the parameters c = 6 and m = 1.25 [80].

The transcriptomics data for the *ache* and *hsp90a−/−* mutants and the corresponding wild-type siblings at 72 hpf were generated as described before on two lanes of 2 × 50 bp in duplicate or triplicate (330 millions read pairs), and aligned to the reference genome as before with TopHat (Additional file 1). The data from *unc45b*, *ache* and *hsp90a* mutants at 72 hpf were analyzed with DESeq2, which have a better power of analysis compared with DESeq and thus detect more misexpressed genes. The robustness of the biological replicates is shown on the heatmap of Euclidean distances in Figure S1a in Additional file 2 (right panel). At a global level the analysis of the *unc45b* data at 72 hpf by DESeq and DESeq2 correlate well, with a Pearson correlation coefficient of the log2 fold change *r* = 0.82, showing the similarity between the two methods.

GO term enrichment studies were carried out on gene clusters obtained by clustering using Metacore (Thomson Reuters) or by calculating *p* values from the Fisher's exact test. For this purpose human orthologs were obtained from Ensembl Compara to query GO terms and process pathways significantly enriched in the different clusters. For the scanning of TF binding sites in the genes obtained by fuzzy mean clustering, promoters including −1 kb relative to the transcriptional start site were analyzed by Opossum [81] and Pscan [82]. Human orthologs for each cluster were obtained from Biomart and −1 kb sequence from the transcriptional start site scanned as before. To search for the Hsf1 binding site in regulatory sequences of the 88 upregulated TFs (FDR < 0.1 and fold change > 1.5), −1 kb to +1 kb to the transcriptional start site was scanned with Opposum v.3.0. *P* values were computed from raw Z-scores obtained from Opposum (*p* < 10^−20^ and Z-score > 10 were considered as significant).

### Ethical approval

Experiments on animals were performed in accordance with the German animal protection standards and were approved by the Government of Baden-Württemberg, Regierungspräsidium Karlsruhe, Germany (Aktenzeichen 35-9185.81/G-137/10").

## Availability of supporting data

The data set supporting the results of this article are available in the Gene Expression Omnibus repository, accession number [GEO:GSE74202] (http://www.ncbi.nlm.nih.gov/geo/query/acc.cgi?acc=GSE74202).

## Additional files

Additional file 1: Table S1. Quality of sequences obtained with CASAVA 1.8.1 (Illumina) workflow. *PF* reads passing Illumina chastity filter. (XLSX 46 kb) Additional file 2: Figure S1.
**a**, **b** Heatmap of Euclidian distance of RNASeq experiments. Pairwise comparison of conditions is displayed as a matrix of Euclidean distances with *red colors* indicating similarities (short distance) and *yellow* dissimilarities (high distance) between conditions. **a** RNA was prepared from either wild-type siblings (*C1* and *C2*) or *unc45b* mutants (*U*) at 24, 48 and 72 hpf. Biological repeats are indicated by the numbers (1–3). *C* control, *U unc45* ^*−/−*^ *.* **b** Clustering of Euclidean distances of the *unc45b*, *hsp90a* and *ache* mutants and their respective wild-type siblings at 72 hpf. **c–e** Fold-change expression of selected genes between wild-type and *unc45b* mutant embryos at 24, 48 and 72 hpf as detected in the RNASeq data. The expression of *unc45b* (**c**), *hsp90a* (**d**) and *smyd1b* (**e**) mRNA steadily increases from 24–72 hpf. **f**, **g** Log2 fold change of genes found upregulated at 72 hpf in *unc45b* and *hsp90a* mutants but not in *ache* mutants (**f**), or upregulated in the three mutants (**g**). Note that the level of upregulation in *ache* mutant is really low compared with the *unc45b* and *hsp90a* mutants. **h**–**j** MA plots displaying log_10_ (mean expression) between control and *unc45b* ^*−/−*^ mutants against log_2_ (fold change) at three developmental stages obtained with DESeq: 24 hpf (**h**), 48 hpf (**i**), 72 hpf (**j**). Genes up- or down-regulated with FDR < 0.05 and fold change > 1.5 are shown in *red*. Numbers indicate the total number of detected genes with FDR < 0.05 and fold change > 1.5. The error bars indicate "standard deviation". (PDF 3965 kb) Additional file 3: Table S2. The 1411 genes regulated in the *unc45b* mutant compared with wild-type embryos at 72 hpf with FDR < 0.05 and fold change > 1.5. (XLSX 120 kb) Additional file 4: Table S3. GO term enrichment analysis of genes up- or down-regulated in *unc45b* mutants compared with wild type at 72 hpf. Cluster groups (1 and 2) were obtained from the hierarchical clustering of the 1411 genes regulated at 72 hpf (Additional file 3). (XLSX 43 kb) Additional file 5: Table S4. Complete list of the 1825 genes regulated in at least one condition (FDR ≤ 0.05). Normalized count, fold change and adjusted *p* values were obtained using the DESeq algorithm. Cluster numbers from fuzzy mean clustering (last column) were assigned for membership > 0.5. (XLSX 225 kb) Additional file 6: Table S5. GO term enrichment analysis of genes in each cluster obtained from fuzzy mean clustering. Cluster numbers are indicated as well as the enriched pathways, FDR and gene name. (XLSX 34 kb) Additional file 7: Figure S2. Chart representing the mean TFP or GFP intensity of transgenic embryos. **a** Mean pixel intensity of *tg(−1.8unc45b:tfp)* embryos compared with *tg(−1.8unc45b:tfp)* injected with either *unc45b-mo* or *hsp90a-mo*, showing twice as much TFP fluorescence in the morphant compared with wild-type embryos. **b** Mean pixel intensity of *tg(−1.8unc45b:tfp)* embryos compared with *tg(−1.8unc45b:tfp) unc45b* mutant showing twice as much TFP fluorescence in the mutant compared with the wild-type embryos. **c** Mean pixel intensity of *tg(−505/−310(unc45b)gata2:gfp*) embryos compared with *tg(−505/−310(unc45b)gata2:gfp)* injected with *hsp90a-mo*. The GFP intensity in the morphant is increased six times compared with wild type. **d** Mean pixel intensity of *tg(−505/−310(unc45b)gata2:gfp)* embryos compared to *tg(−505/−310(unc45b)gata2:gfp)* injected with *unc45b-mo* or *unc45b-mo* and *hsf1-mo*. The *hsf1-mo* abolished the increased GFP expression obtained with *unc45b-mo* alone. **e** Mean pixel intensity of *tg(−505/−310(unc45b)gata2:gfp)* embryos compared with *tg(−505/−310(unc45b)gata2:gfp)* injected with *unc45b-mo* or *unc45b-mo* and *hsf2-mo*. The *hsf2-mo* does not reduce the increased GFP expression obtained with *unc45b-mo* alone. **f** Mean pixel intensity of *tg(−505/−310(unc45b)gata2:gfp)* embryos compared with *tg(−505/−310(unc45b)gata2:gfp)* injected with *unc45b-mo* or *unc45b-mo* and *hsf1cont-mo*. The *hsf1cont-mo* does not reduce the increased GFP expression obtained with *unc45b-mo* alone. For each measurement at least three embryos were examined. For each chart the measured embryos are progeny of homozygous transgenics crossed with wild-type adult fish, and were exposed to the same light intensity. The error bars indicate "standard deviation". (PDF 498 kb) Additional file 8: Figure S3. Summary of the transgenes used to map the regulatory elements mediating the misfolded myosin response A-B: tg(-1.8unc45b:tfp) (“1”) and its deletion derivatives were expressed transiently (trs) or as transgenesstably integrated into the genome (stb). By serial deletion analysis 645 bp upstream of the ATG of unc45bwas identified to still drive basal muscle expression and respond to unc45b deficiency (B). Furthertruncation of the unc45b sequences to the region from -369 bp to +390 bp abolished the response.C-E: Refined analysis of the unc45b regulatory sequences contained in tg(-645/+533unc45b:tfp). Theunc45b deletion fragments were cloned in front of the gata2 promoter (blue bars). Transgenes containingthe 195 bp fragment (“16”, D) harboring sequences from -505 to -310 drove GFP expression in skeletalmuscle (D, cont) and reacted to the accumulation of unfolded myosin (D, +unc45b-mo). Deletion constructs12-20 retained basal muscle expression and responded to misfolded myosin. In contrast, constructs 21-24give no GFP expression (see E).Further mutations of the 195 bp unc45b fragment separates the regulatory sequences responsible for basalmuscle expression and the response to misfolded myosin. Construct 28 drove basal GFP expression inskeletal muscles but did not respond when co-injected with the unc45b-mo (G). Construct 30 (55 bp)reacted similarly (H). These constructs retained a binding site of the Mef2 transcription factor. Constructs 31-37 lack the muscle basal expression but still respond to misfolded myosin (I). Thus, the region from -445 to -425 is important to mediate the response. This region contains a homology to the heat shock responseelement (HSE, J). The HSE was mutated by introduction of 4 point mutations (asterisks J, F, I) in constructs“26”, “38”, “41” and “42”. Mutation of the HSE leads to loss of the misfolded myosin response. Abbreviations:ME: muscle expression. The numbers above constructs indicate position relative to the ATG of unc45b. (PDF 2497 kb) Additional file 9: Figure S4.
**a**, **b** Sequence comparison of the *unc45b* region mediating the misfolded myosin response with homologous regions in the *unc45b* genes of other fish species. **a** Scheme of the 195-bp zebrafish *unc45b* fragment (−505 to −310 relative to the ATG) containing the HSE at position −446/−422 and the Mef2 binding site at position −335/−313. The recognition sequence is depicted below each site. **b** Comparison of the zebrafish *unc45b* sequence from −505 to −310 with regions in the *unc45b* genes of four other fish species revealed the presence of a conserved HSE element (*black ovals*). **c–e** Injection of −*505/*−*405(unc45b)gata2:gfp* plasmid into wild-type embryos (*cont*) (**c**) do not generate any GFP expression, whereas co-injection with *unc45b-mo* activates the regulatory sequence (**c**) (*unc45b-mo*). Examination of the GFP fibrils revealed classic striations (**d**, **e**, **z**) (Z-line). **f** injection of −*505/*−*405(unc45b)gata2:gfp* plasmid into *hel* mutant (*hel−/−*) or wild-type siblings (*cont*) do not lead to GFP0 expression. **g**, **h** Injection of −*505/*−*310(unc45b)gata2:gfp* plasmid into wild type (*cont*) (**g**, **h**), *unc45b* morphant (*unc45b-mo*) (**g**) or *hel* mutants (*hel−/−*) (**h**) show a GFP upregulation in *unc45b* morphant but not in *hel* mutants. (PDF 3322 kb) Additional file 10: Figure S5.
**a** *Tg(−505/−310 (unc45b)gata2:gfp)* embryos were injected with *hsp90a* morpholinos (*hsp90a-mo*) or with *hsp90a-mo* together with a mix of CRISPR RNA directed against *hsf1* and *cas9* mRNA (*hsp90a-mo + guide hsf1*). We note a decrease of GFP in the *hsf1*/*hsp90a* double knock-down compared with the *hsp90a* single morphant. **b**–**b’** Injection of the plasmid encoding a Hsf1–mOrange fusion protein does not activate the *unc45b* promoter. Expression of TFP reporter in *hsf1-morange* injected embryos (+hsf1:morange) (**a**, **b**) is as high as in uninjected *tg(−1.8unc45b:tfp)* embryos (*cont*) (**a**). **c**–**h’** Heat shock triggers *unc45b* up-regulation. *−505/−310(unc45b)gata2:gfp* injected embryos (**c**, **d**, **c’**, **d’**), *Tg(−505/−310 (unc45b)gata2:gfp)* (**e, f, e’, f’**) and *tg(−1.8unc45b:tfp)* (**g**, **h**, **g’**, **h’**) transgenic embryos were raised at 28 °C until 48 hpf and heat shock at 37 °C for 12 hours (**d**, **d’**, **f**, **f’**, **h**, **h’**). Embryos shown in (**c**, **c’**, **e**, **e**, **g**, **g’**) were kept at 28 °C. The heat shock embryos show up-regulation of GFP in skeletal and cardiac muscles, demonstrating that the regulatory sequences of *unc45b* react to heat shock. (PDF 1668 kb) Additional file 11: Figure S6.
**a** Plot of Z-score versus percentage GC content for transcription factor binding sites detected in promoter regions of zebrafish genes belonging to cluster 4 (n = 178 genes). The *red dashed line* defines background level (Z- score > mean + 2 standard deviations). **b** Detection of Hsf1 binding site in −1 kb relative to transcription start site of human orthologous genes from each cluster obtained by fuzzy mean clustering. The number of human orthologs found is indicated for each cluster. Asterisks indicate significant enrichment of Hsf1 binding sites. **c** Example of genome browser views of human HSF1 ChIP-Seq data for four genes belonging to cluster 4. Gene name, structure and directionality are indicated for each gene, as well as HSF1 (*pink*) and H3K27Ac (*black*) chromatin immunoprecipitation tracks. Hsf1 binding sites are indicated by *red arrows*. (PDF 546 kb) Additional file 12: Table S6. Genes and positions of Hsf1 binding sites. Gene name and sequence information are indicated for each gene from clusters 1 and 4 (from fuzzy mean clustering) with a detected Hsf1 binding site. Scores are obtained with the Opposum v.3.0 algorithm. (XLSX 45 kb) Additional file 13: Table S7. Full list of Hsf1 binding sites detected in the promoter of genes up-regulated concomitantly in *unc45b* −/− and *hsp90a* −/− compared with wild type at 72 hpf. Gene name and sequence information are indicated for each gene with a detected Hsf1 binding site. Scores are obtained with the Opposum v.3.0 algorithm. (XLSX 50 kb)

## Abbreviations

bp: base pair
FDR: false discovery rate
GFP: green fluorescent protein
GO: gene ontology
hpf: hours post-fertilization
HSE: heat shock element
Hsf: Heat shock factor
TF: transcription factor
TFP: teal fluorescent protein
