# Supplementary figures and images for: Loss of function of myosin chaperones triggers Hsf1-mediated transcriptional response in skeletal muscle cells
Source: Genome Biol. 2015 Dec 3;16:267. doi: 10.1186/s13059-015-0825-8 (PMC4668643; doi:10.1186/s13059-015-0825-8)

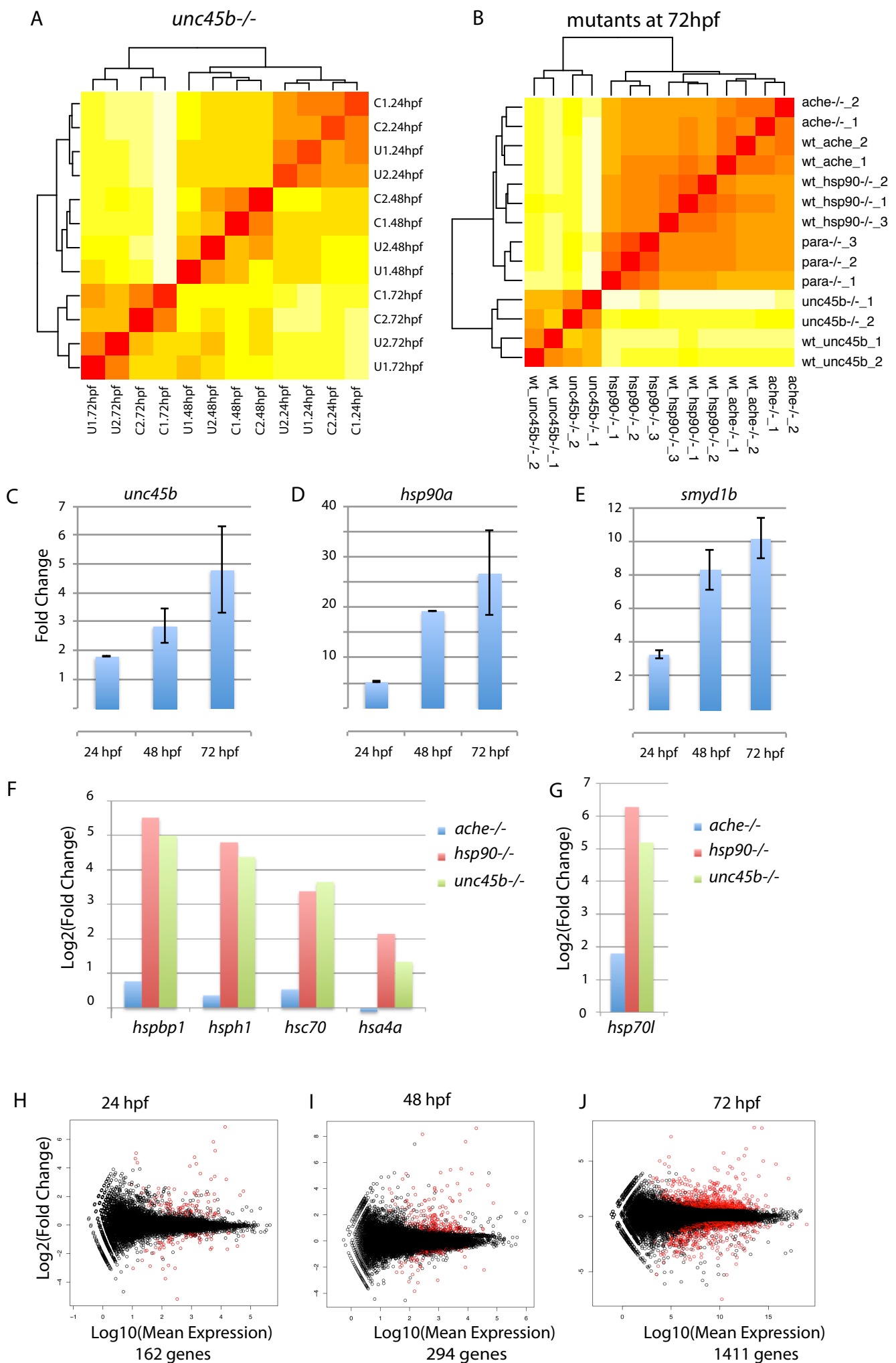

Supplement: Additional file 2: Figure S1. — a, b Heatmap of Euclidian distance of RNASeq experiments. Pairwise comparison of conditions is displayed as a matrix of Euclidean distances with red colors indicating similarities (short distance) and yellow dissimilarities (high distance) between conditions. a RNA was prepared from either wild-type siblings (C1 and C2) or unc45b mutants (U) at 24, 48 and 72 hpf. Biological repeats are indicated by the numbers (1–3). C control, U unc45 −/− . b Clustering of Euclidean distances of the unc45b, hsp90a and ache mutants and their respective wild-type siblings at 72 hpf. c–e Fold-change expression of selected genes between wild-type and unc45b mutant embryos at 24, 48 and 72 hpf as detected in the RNASeq data. The expression of unc45b (c), hsp90a (d) and smyd1b (e) mRNA steadily increases from 24–72 hpf. f, g Log2 fold change of genes found upregulated at 72 hpf in unc45b and hsp90a mutants but not in ache mutants (f), or upregulated in the three mutants (g). Note that the level of upregulation in ache mutant is really low compared with the unc45b and hsp90a mutants. h–j MA plots displaying log10 (mean expression) between control and unc45b −/− mutants against log2 (fold change) at three developmental stages obtained with DESeq: 24 hpf (h), 48 hpf (i), 72 hpf (j). Genes up- or down-regulated with FDR < 0.05 and fold change > 1.5 are shown in red. Numbers indicate the total number of detected genes with FDR < 0.05 and fold change > 1.5. The error bars indicate "standard deviation". (PDF 3965 kb) [file 13059_2015_825_MOESM2_ESM.pdf]

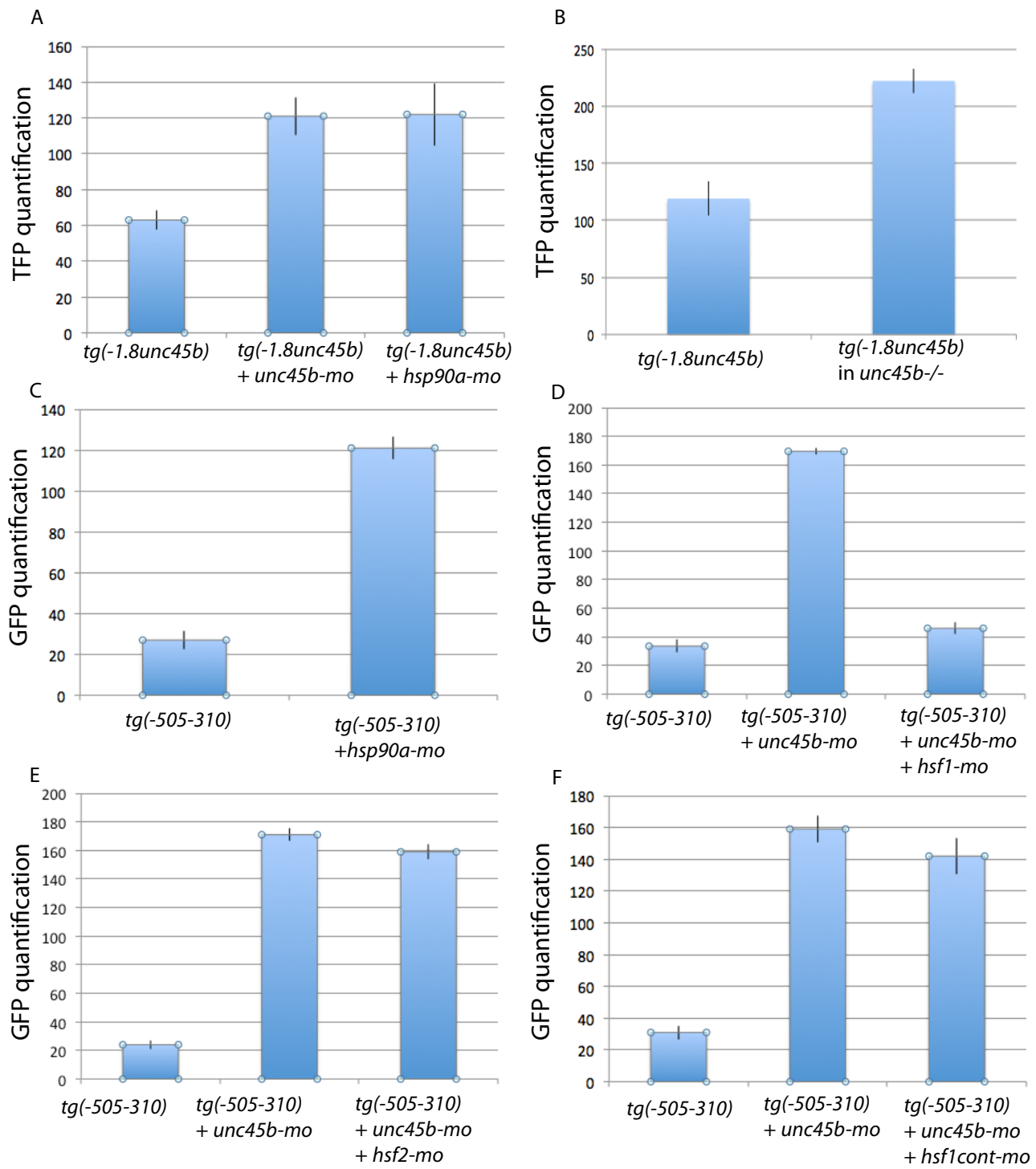

Etard et al., Suppl. Fig. 2

Supplement: Additional file 7: Figure S2. — Chart representing the mean TFP or GFP intensity of transgenic embryos. a Mean pixel intensity of tg(−1.8unc45b:tfp) embryos compared with tg(−1.8unc45b:tfp) injected with either unc45b-mo or hsp90a-mo, showing twice as much TFP fluorescence in the morphant compared with wild-type embryos. b Mean pixel intensity of tg(−1.8unc45b:tfp) embryos compared with tg(−1.8unc45b:tfp) unc45b mutant showing twice as much TFP fluorescence in the mutant compared with the wild-type embryos. c Mean pixel intensity of tg(−505/−310(unc45b)gata2:gfp) embryos compared with tg(−505/−310(unc45b)gata2:gfp) injected with hsp90a-mo. The GFP intensity in the morphant is increased six times compared with wild type. d Mean pixel intensity of tg(−505/−310(unc45b)gata2:gfp) embryos compared to tg(−505/−310(unc45b)gata2:gfp) injected with unc45b-mo or unc45b-mo and hsf1-mo. The hsf1-mo abolished the increased GFP expression obtained with unc45b-mo alone. e Mean pixel intensity of tg(−505/−310(unc45b)gata2:gfp) embryos compared with tg(−505/−310(unc45b)gata2:gfp) injected with unc45b-mo or unc45b-mo and hsf2-mo. The hsf2-mo does not reduce the increased GFP expression obtained with unc45b-mo alone. f Mean pixel intensity of tg(−505/−310(unc45b)gata2:gfp) embryos compared with tg(−505/−310(unc45b)gata2:gfp) injected with unc45b-mo or unc45b-mo and hsf1cont-mo. The hsf1cont-mo does not reduce the increased GFP expression obtained with unc45b-mo alone. For each measurement at least three embryos were examined. For each chart the measured embryos are progeny of homozygous transgenics crossed with wild-type adult fish, and were exposed to the same light intensity. The error bars indicate "standard deviation". (PDF 498 kb) [file 13059_2015_825_MOESM7_ESM.pdf]

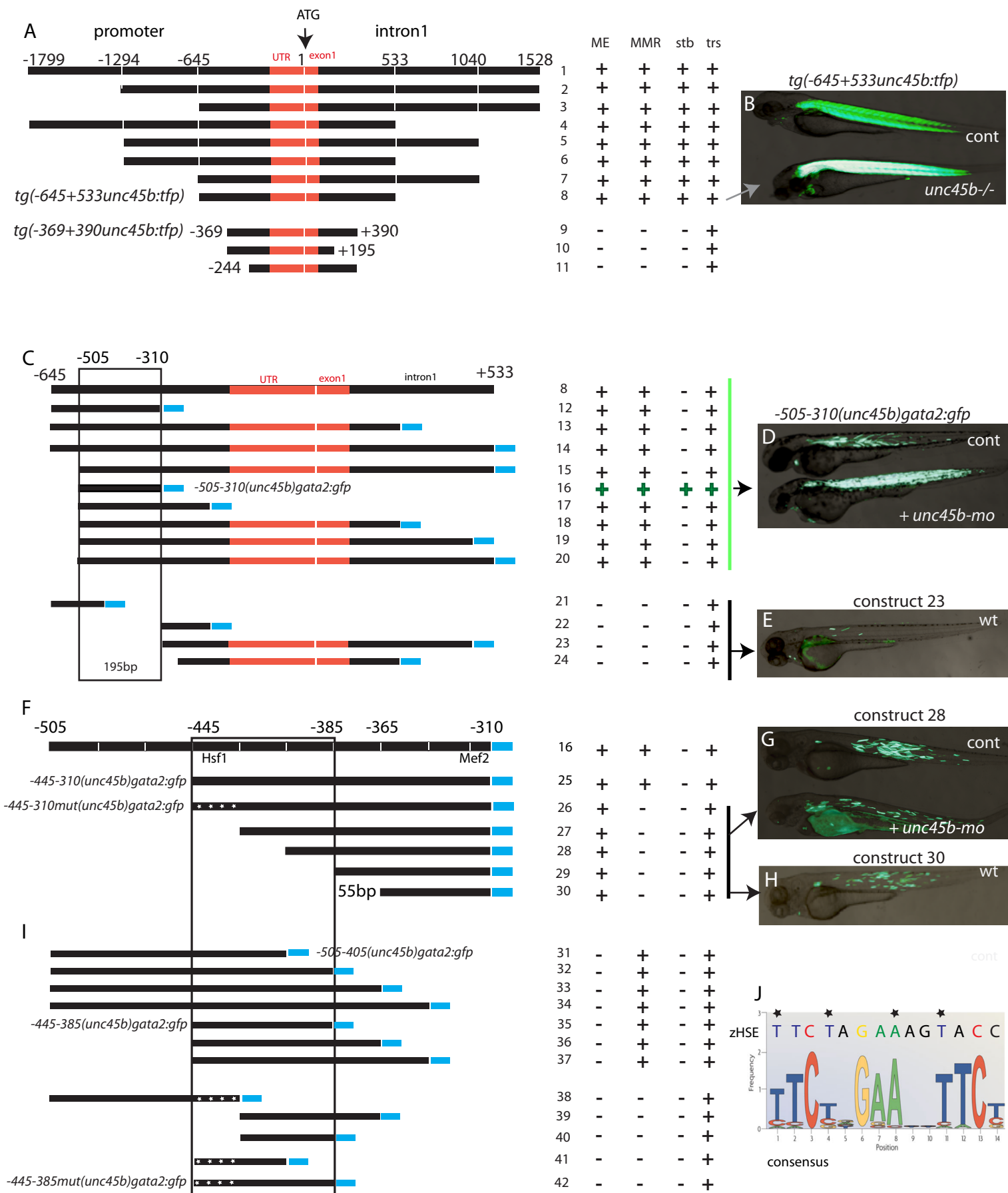

Etard et al., Suppl. Fig. 3

Supplement: Additional file 8: Figure S3. — Summary of the transgenes used to map the regulatory elements mediating the misfolded myosin response A-B: tg(-1.8unc45b:tfp) (“1”) and its deletion derivatives were expressed transiently (trs) or as transgenesstably integrated into the genome (stb). By serial deletion analysis 645 bp upstream of the ATG of unc45bwas identified to still drive basal muscle expression and respond to unc45b deficiency (B). Furthertruncation of the unc45b sequences to the region from -369 bp to +390 bp abolished the response.C-E: Refined analysis of the unc45b regulatory sequences contained in tg(-645/+533unc45b:tfp). Theunc45b deletion fragments were cloned in front of the gata2 promoter (blue bars). Transgenes containingthe 195 bp fragment (“16”, D) harboring sequences from -505 to -310 drove GFP expression in skeletalmuscle (D, cont) and reacted to the accumulation of unfolded myosin (D, +unc45b-mo). Deletion constructs12-20 retained basal muscle expression and responded to misfolded myosin. In contrast, constructs 21-24give no GFP expression (see E).Further mutations of the 195 bp unc45b fragment separates the regulatory sequences responsible for basalmuscle expression and the response to misfolded myosin. Construct 28 drove basal GFP expression inskeletal muscles but did not respond when co-injected with the unc45b-mo (G). Construct 30 (55 bp)reacted similarly (H). These constructs retained a binding site of the Mef2 transcription factor. Constructs 31-37 lack the muscle basal expression but still respond to misfolded myosin (I). Thus, the region from -445 to -425 is important to mediate the response. This region contains a homology to the heat shock responseelement (HSE, J). The HSE was mutated by introduction of 4 point mutations (asterisks J, F, I) in constructs“26”, “38”, “41” and “42”. Mutation of the HSE leads to loss of the misfolded myosin response. Abbreviations:ME: muscle expression. The numbers above constructs indicate position relative to the ATG of unc45b. (PDF 2497 k [file 13059_2015_825_MOESM8_ESM.pdf]

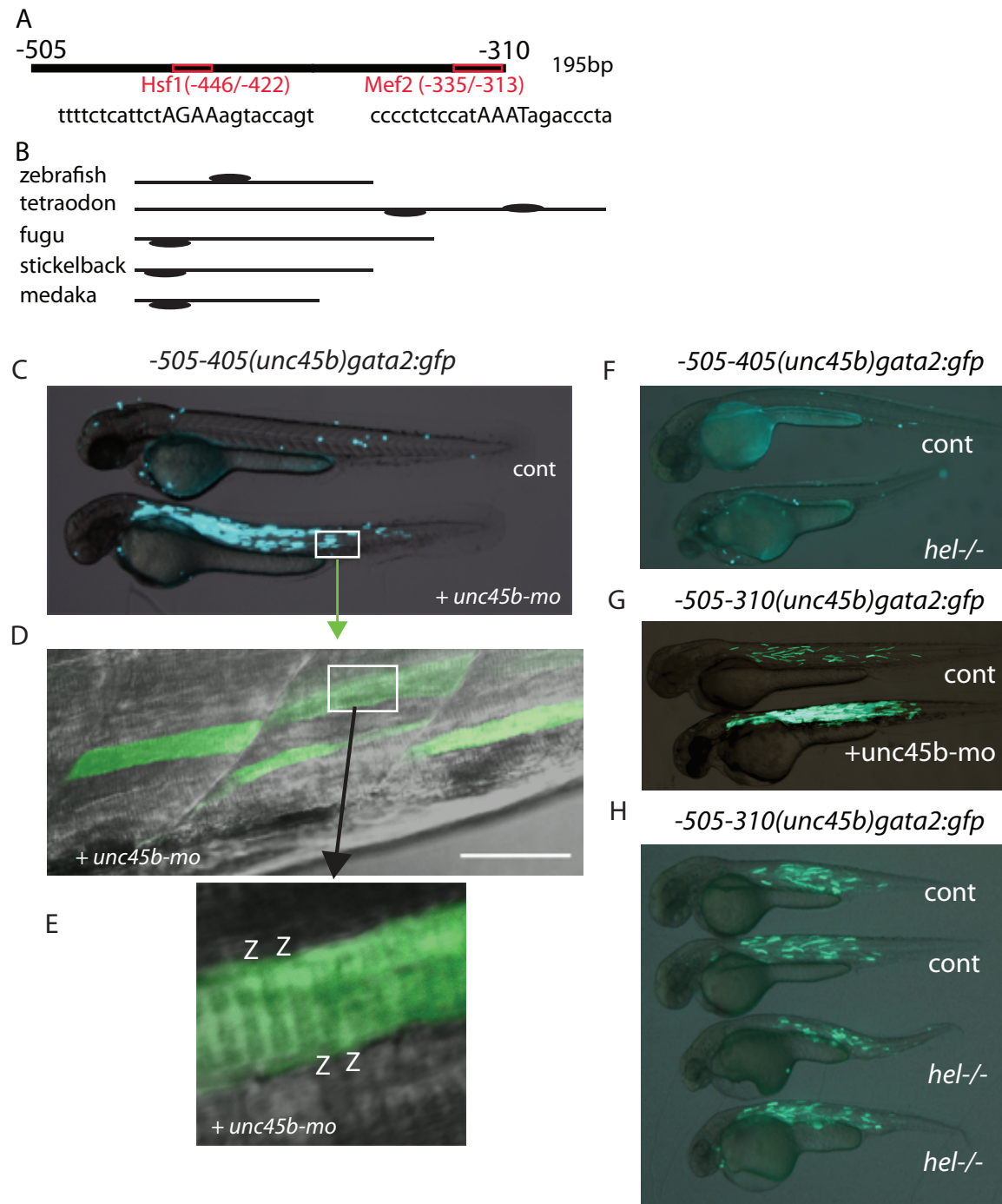

Etard et al., Suppl. Fig. 4

Supplement: Additional file 9: Figure S4. — a, b Sequence comparison of the unc45b region mediating the misfolded myosin response with homologous regions in the unc45b genes of other fish species. a Scheme of the 195-bp zebrafish unc45b fragment (−505 to −310 relative to the ATG) containing the HSE at position −446/−422 and the Mef2 binding site at position −335/−313. The recognition sequence is depicted below each site. b Comparison of the zebrafish unc45b sequence from −505 to −310 with regions in the unc45b genes of four other fish species revealed the presence of a conserved HSE element (black ovals). c–e Injection of −505/−405(unc45b)gata2:gfp plasmid into wild-type embryos (cont) (c) do not generate any GFP expression, whereas co-injection with unc45b-mo activates the regulatory sequence (c) (unc45b-mo). Examination of the GFP fibrils revealed classic striations (d, e, z) (Z-line). f injection of −505/−405(unc45b)gata2:gfp plasmid into hel mutant (hel−/−) or wild-type siblings (cont) do not lead to GFP0 expression. g, h Injection of −505/−310(unc45b)gata2:gfp plasmid into wild type (cont) (g, h), unc45b morphant (unc45b-mo) (g) or hel mutants (hel−/−) (h) show a GFP upregulation in unc45b morphant but not in hel mutants. (PDF 3322 kb) [file 13059_2015_825_MOESM9_ESM.pdf]

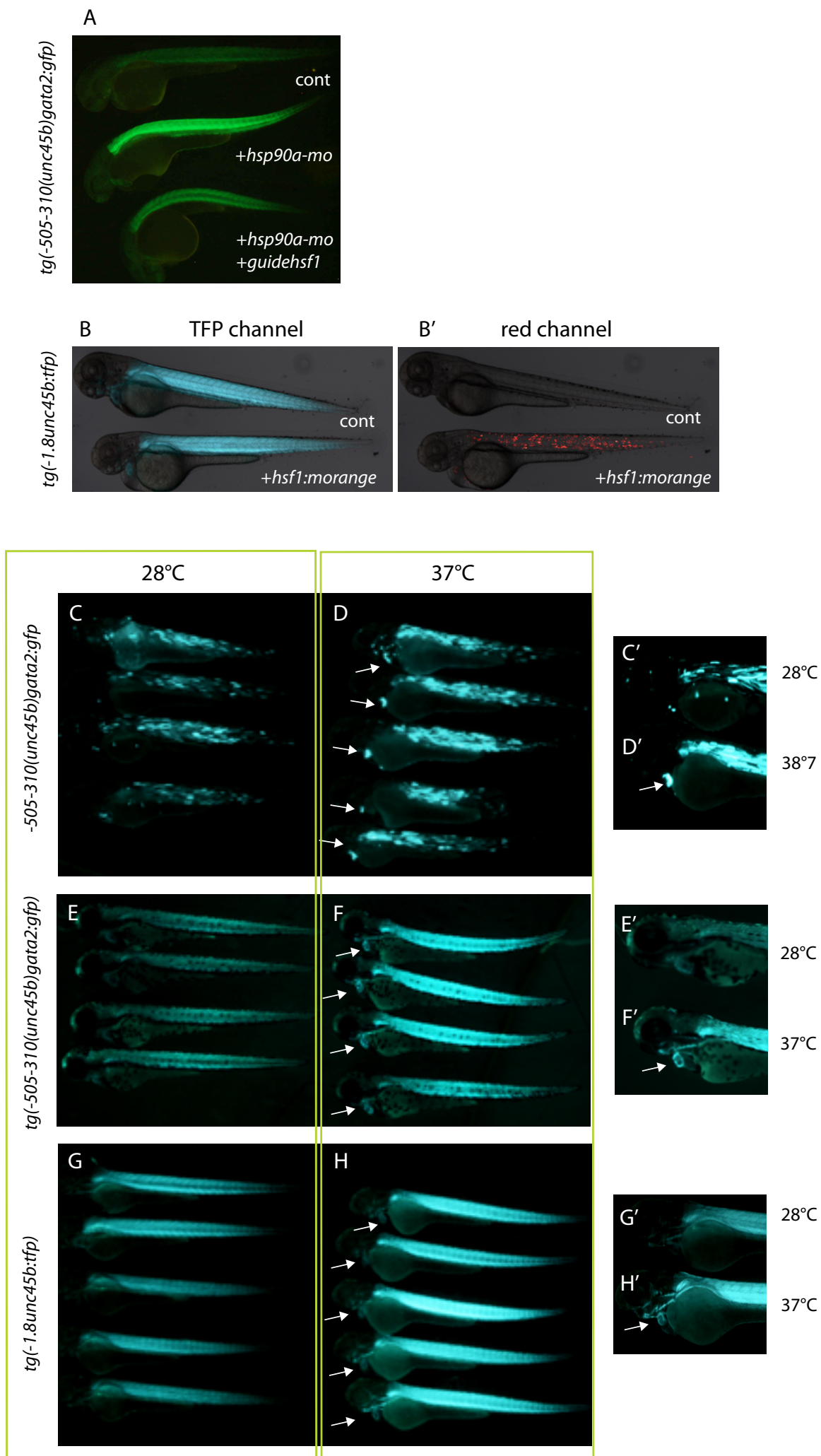

Supplement: Additional file 10: Figure S5. — a Tg(−505/−310 (unc45b)gata2:gfp) embryos were injected with hsp90a morpholinos (hsp90a-mo) or with hsp90a-mo together with a mix of CRISPR RNA directed against hsf1 and cas9 mRNA (hsp90a-mo + guide hsf1). We note a decrease of GFP in the hsf1/hsp90a double knock-down compared with the hsp90a single morphant. b–b’ Injection of the plasmid encoding a Hsf1–mOrange fusion protein does not activate the unc45b promoter. Expression of TFP reporter in hsf1-morange injected embryos (+hsf1:morange) (a, b) is as high as in uninjected tg(−1.8unc45b:tfp) embryos (cont) (a). c–h’ Heat shock triggers unc45b up-regulation. −505/−310(unc45b)gata2:gfp injected embryos (c, d, c’, d’), Tg(−505/−310 (unc45b)gata2:gfp) (e, f, e’, f’) and tg(−1.8unc45b:tfp) (g, h, g’, h’) transgenic embryos were raised at 28 °C until 48 hpf and heat shock at 37 °C for 12 hours (d, d’, f, f’, h, h’). Embryos shown in (c, c’, e, e, g, g’) were kept at 28 °C. The heat shock embryos show up-regulation of GFP in skeletal and cardiac muscles, demonstrating that the regulatory sequences of unc45b react to heat shock. (PDF 1668 kb) [file 13059_2015_825_MOESM10_ESM.pdf]

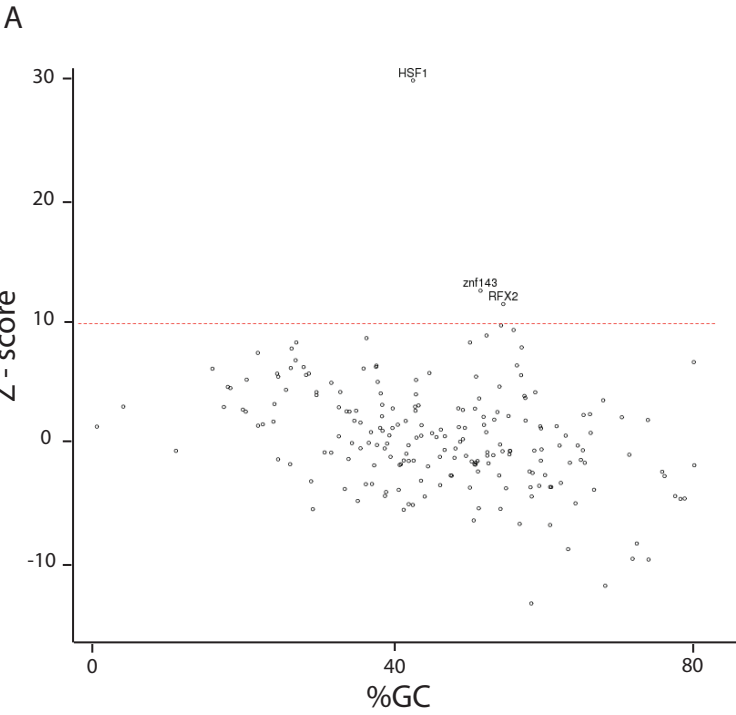

**B**

| Cluster | Total Human orthologues | p-value   |
|---------|-------------------------|-----------|
| 1       | 135                     | 4.9E-01   |
| 2       | 162                     | 9.9E-01   |
| 3       | 137                     | 5.3E-01   |
| 4       | 153                     | 1.3E-04 * |
| 5       | 338                     | 9.0E-01   |
| 6       | 250                     | 9.3E-01   |

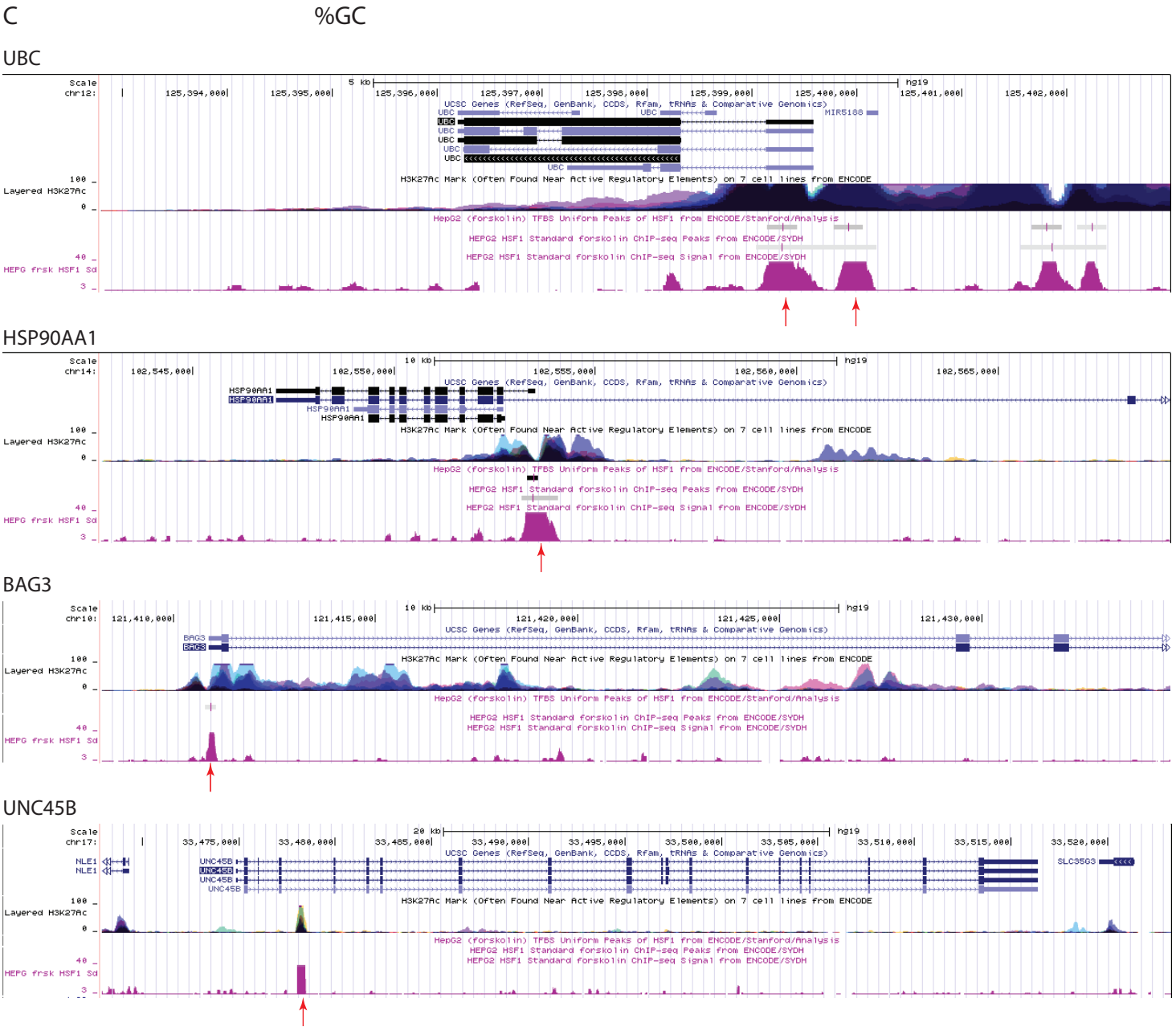

Supplement: Additional file 11: Figure S6. — a Plot of Z-score versus percentage GC content for transcription factor binding sites detected in promoter regions of zebrafish genes belonging to cluster 4 (n = 178 genes). The red dashed line defines background level (Z- score > mean + 2 standard deviations). b Detection of Hsf1 binding site in −1 kb relative to transcription start site of human orthologous genes from each cluster obtained by fuzzy mean clustering. The number of human orthologs found is indicated for each cluster. Asterisks indicate significant enrichment of Hsf1 binding sites. c Example of genome browser views of human HSF1 ChIP-Seq data for four genes belonging to cluster 4. Gene name, structure and directionality are indicated for each gene, as well as HSF1 (pink) and H3K27Ac (black) chromatin immunoprecipitation tracks. Hsf1 binding sites are indicated by red arrows. (PDF 546 kb) [file 13059_2015_825_MOESM11_ESM.pdf]
